# Phylodynamic Analysis Reveals CRF01_AE Dissemination between Japan and Neighboring Asian Countries and the Role of Intravenous Drug Use in Transmission

**DOI:** 10.1371/journal.pone.0102633

**Published:** 2014-07-15

**Authors:** Teiichiro Shiino, Junko Hattori, Yoshiyuki Yokomaku, Yasumasa Iwatani, Wataru Sugiura, Japanese Drug Resistance HIV-1 Surveillance Network

**Affiliations:** 1 Infectious Disease Surveillance Center, National Institute of Infectious Diseases, Tokyo, Japan; 2 Department of Infectious Diseases and Immunology, Clinical Research Center, Nagoya Medical Center, Nagoya, Japan; 3 Department of AIDS Research, Nagoya University Graduate School of Medicine, Nagoya, Japan; Centers for Disease Control and Prevention, United States of America

## Abstract

**Background:**

One major circulating HIV-1 subtype in Southeast Asian countries is CRF01_AE, but little is known about its epidemiology in Japan. We conducted a molecular phylodynamic study of patients newly diagnosed with CRF01_AE from 2003 to 2010.

**Methods:**

Plasma samples from patients registered in Japanese Drug Resistance HIV-1 Surveillance Network were analyzed for protease-reverse transcriptase sequences; all sequences undergo subtyping and phylogenetic analysis using distance-matrix-based, maximum likelihood and Bayesian coalescent Markov Chain Monte Carlo (MCMC) phylogenetic inferences. Transmission clusters were identified using interior branch test and depth-first searches for sub-tree partitions. Times of most recent common ancestor (tMRCAs) of significant clusters were estimated using Bayesian MCMC analysis.

**Results:**

Among 3618 patient registered in our network, 243 were infected with CRF01_AE. The majority of individuals with CRF01_AE were Japanese, predominantly male, and reported heterosexual contact as their risk factor. We found 5 large clusters with ≥5 members and 25 small clusters consisting of pairs of individuals with highly related CRF01_AE strains. The earliest cluster showed a tMRCA of 1996, and consisted of individuals with their known risk as heterosexual contacts. The other four large clusters showed later tMRCAs between 2000 and 2002 with members including intravenous drug users (IVDU) and non-Japanese, but not men who have sex with men (MSM). In contrast, small clusters included a high frequency of individuals reporting MSM risk factors. Phylogenetic analysis also showed that some individuals infected with HIV strains spread in East and South-eastern Asian countries.

**Conclusions:**

Introduction of CRF01_AE viruses into Japan is estimated to have occurred in the 1990s. CFR01_AE spread via heterosexual behavior, then among persons connected with non-Japanese, IVDU, and MSM. Phylogenetic analysis demonstrated that some viral variants are largely restricted to Japan, while others have a broad geographic distribution.

## Introduction

Since the first HIV-1-infected case was identified in Japan in 1985, the cumulative number of reported cases of HIV/AIDS has been increasing every year, reaching 18,447 by the end of 2010 [1]. The major HIV-1 subtype in the 1980s in Japan was subtype B [2] followed by CRF01_AE [3]. CRF01_AE caused an outbreak among the high-risk heterosexual population in Thailand in the late 1980s [4]–[6], and was subsequently disseminated to various risk populations in neighboring countries, including Vietnam, Cambodia, Malaysia, Indonesia and China [7]–[15]. Overall the CRF01_AE is substantial, accounting for an estimated 36% of HIV in South, Southeast, and East Asia (Los Alamos database) in CRF01_AE was likely introduced into Japan’s heterosexual population in the early phase of the epidemic [3], [4], [7], but the characteristics of the spread of CRF01_AE in Japan have not been extensively investigated. Our surveillance research showed that from 2003 to 2008 CRF01_AE was the second most prevalent subtype (8.4%) after subtype B, and its host characteristics are distinct from those of the subtype B population [16]. CRF01_AE cases are significantly linked to heterosexual transmission [3], [16], [17] and non-Japanese people [16]. In contrast, subtype B tends to be found in men who have sex with men (MSM) and Japanese people.

CRF01_AE cases appear to be diagnosed in Japan at a later stage of infection [16], and trends in the CRF01_AE epidemic in Japan have been difficult to study by conventional descriptive epidemiological methods. However, recent advances in computational science have allowed us to infer the evolutionary dynamics of a pathogen population from large-scale sequence data using methods, now referred to as “phylodynamics” [18]. Phylodynamics has been used to aid in the analysis of spread of infectious agents with a rapid evolutionary rate [18], e.g., RNA viruses including influenza A [19]–[21], hepatitis C [22], [23], and HIV-1 [24]–[26]. Since 2003, we have been collecting HIV-1 nucleotide sequence data from newly diagnosed patients in Japan as part of our nationwide surveillance project [16], [27]. Here we report our results from applying the phylodynamics approach to these sequence data to understand trends in the CRF01_AE outbreak in Japan, genetic relationships between the circulating strains within Japan and strains observed in the surrounding Asian countries, details of their transmission risk factors, and finally to identify the target populations for effective action plans to prevent further transmission of CRF01_AE.

## Materials and Methods

### Ethical Statement

This study was conducted according to principles in the Declaration of Helsinki. The study was approved by the human subject research committee at the National Institute of Infectious Diseases and Nagoya Medical Center, Japan. All patients provided written informed consent for the collection of samples and subsequent analyses.

### Sample Collection and Viral Gene Sequences

Viral samples were collected from HIV-1-infected patients newly diagnosed from January 2003 to March 2010 at 30 clinics and public health centers in Japan that have participated in our Japanese Drug Resistance HIV-1 Surveillance Network [16], [27]. These collection areas are classified into 8 regions according to the nationwide systemic network of hospitals in Japan [28] (Figure S1). The study sample comprised 3618 individuals both acutely and chronically infected. At diagnosis or the earliest hospital visit, patients’ peripheral blood was drawn into a vacutainer with EDTA added. At the same time, demographic information was collected on age, gender, nationality, and risk behavior. Plasma samples were analyzed for the nucleotide sequences of HIV-1 protease and the 1- to 240-amino acid region of reverse-transcriptase (RT) using the direct sequencing method of RT-PCR products, and the HIV-1 subtype was determined using phylogenetic analysis as reported [16], [27]. This analysis showed that 243 individuals were infected with CRF01_AE at least in the protease-RT regions. GenBank accession numbers of the nucleotide sequences are AB356098–AB556499, AB44228–AB442360, and AB863746–AB871315.

### Sequence Alignment of Reconstructed Variants

Direct sequence data may contain loci with multiple peaks, including drug-resistance mutation sites. As these ambiguous loci are generally excluded from analysis by phylogenetic programs, and we wanted to use collected sequences to the maximum in further analyses, we separated these multiple nucleotides into individual nucleotides and reconstructed hypothetical sequence variants possessing each nucleotide as follows. Briefly, protease and RT sequences were concatenated and aligned using Clustal W, version 2.0.10 [29]. Then, a consensus sequence was calculated for the alignment, and ambiguous nucleotides were classified into two groups: overlapping and non-overlapping ambiguities. The former shows one polymorphic nucleotide shared with the consensus allele, while the latter shows a fixation of different alleles from the consensus one [26]. The consensus allele found in the overlapping site was adopted for the reconstructed sequence, and the non-overlapping site was segregated into two haplotypes that carried each nucleotide of the ambiguous site. Consequently, the total number of reconstructed sequences became 297.

These sequences were realigned with foreign CRF01_AE outlier sequences selected as follows. Since transmission clusters have been developed according to scale-free networks in many infectious agents [24], [26], [30], [31], we calculated a frequency distribution of the cluster scale-free network for our observed population in Japan (n = 297) by Barabasi’s model of scale-free networks [32]. This calculation used the barabasi.game function in igraph library of R software [33]. We estimated that 32.5% of the sequences should be involved in one cluster. Then, using a one-sided error range and rejection coefficient of 0.025 and 1.96 for within and outside clusters, respectively, we calculated the necessary size of the outlier sequence dataset for even allocations within and outside clusters and out of clusters as greater than 332.1. Based on this estimate, we randomly selected 333 CRF01_AE sequences from 37 countries in five regions (Africa, North America, South America, Asia and Europe) submitted to the Los Alamos HIV database before 2010 and used them as the foreign outlier dataset (Table S1). The sequences were further aligned with the following 6 subtype A outgroup sequences: A1.UG.92.92UG037, A1.KE.94.Q23_17, A1.AU.03.PS1044_Day0, A1.RW.92.92RW008, A2.CD.97.97CDKTB48, and A2.CY.94.94CY017_41. The resulting alignment was corrected by hand for gaps. The reference and outlier sequences were collected from the Los Alamos HIV database (http://www.hiv.lanl.gov/content/index.html). The final number of sites in the aligned sequence was 1150 bases.

### Phylogenetic Inferences of the Viral Gene Sequence

**Figure 1:**
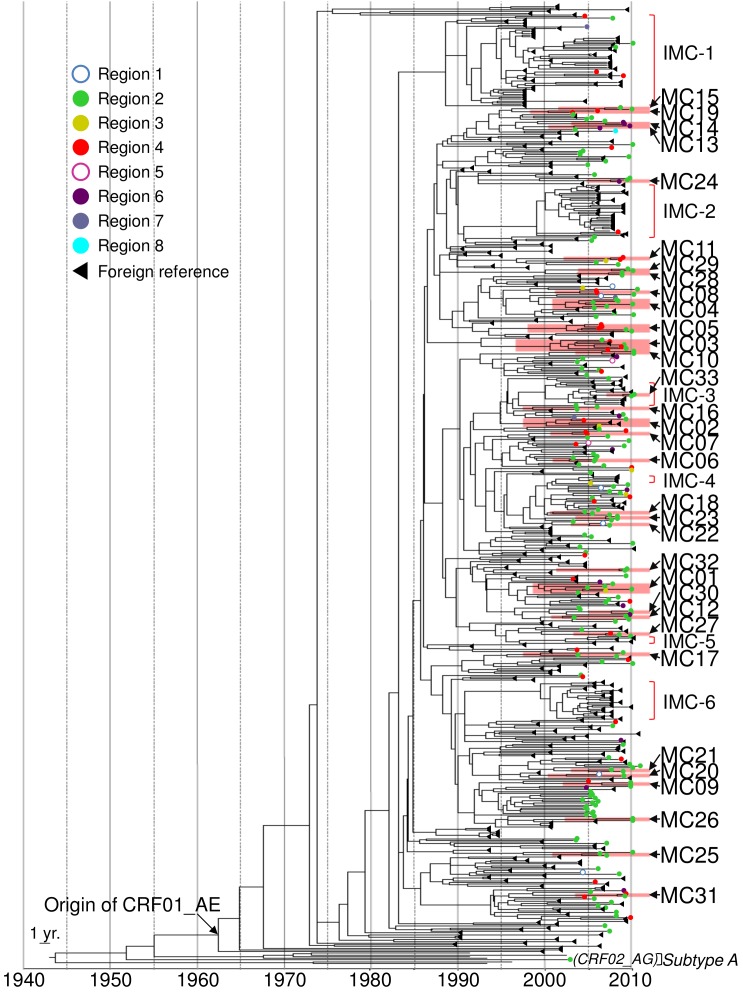
Maximum clade credibility tree for partial *pol* region identifies 33 micro-clades within CRF01_AE cases in Japan. Phylodynamic Analysis. The branch length of the phylogeny is in units of time. Patient sequences obtained from surveillance are designated by open or solid circles. Circle color indicates geographic origin of the samples in Japan. Reference sequences from the Los Alamos HIV database are designated by black triangles. Micro-clades (MC) are annotated by red shading and international micro-clades (IMC) are marked with red brackets on the right of the tree.

To eliminate the influence of antiretroviral drug treatments on viral evolution, we made a codon-stripped sequence alignment by removing 43 drug resistance-associated codons defined in our previous studies [16], [27]. This alignment was used to estimate a matrix of the number of substitutions between each sequence pair by the composite likelihood method [34], and to infer the neighbor-joining (NJ) tree with the interior branch test. The sequence alignment was also used to infer the maximum likelihood tree using the same substitution model described below with a bootstrap test of 500 replicates. In this process, one of the 244 subjects preliminarily classified into CRF01_AE was re-classified into CRF02_AG (Figure 1 and Figure S2). We excluded this subject in the following analyses. The distance matrix was also used to calculate the mean number of base substitutions per site (i.e., genetic diversity) within and between arbitrary subpopulations, and the coefficient of differentiation between subpopulations. Standard error estimates for genetic diversity were obtained by a bootstrap test with 500 replicates. The analyses were conducted using MEGA version 5.0 [35].

### Phylodynamic Analysis

The best-fit model for nucleotide substitution was evaluated by the hierarchical likelihood ratio test using PAUP v4.0 [36] with MrModeltest [37], and the general time-reversible model was adopted with gamma-distributed site heterogeneity and invariant sites (GTR+G+I) with four rate categories. Evolutionary parameters, chronological maximum clade credibility phylogeny, and the times of the most recent common ancestors (tMRCAs) were estimated from the sequence alignment using the Bayesian Coalescent Markov Chain Monte Carlo (MCMC) approach implemented in BEAST v1.7.4 [38]. The sequence was partitioned into 3-codon positions. To select a model for population growth and the molecular clock, we used Bayesian factor comparison [39] with the marginal likelihood estimated by the stepping-stone sampling method [40], [41] using preliminary runs of BEAST with MCMC chains of 100 million iterations. Constant population growth and relaxed clock with an uncorrelated lognormal-distribution was adopted as a best-fit model (Table S2). The best-fit parameters were then used in an additional MCMC analysis consisting of 500 million iterations to estimate the evolutionary parameters. The convergence of parameters was inspected using Tracer v.1.5, with uncertainties depicted as 95% highest probability density (HPD) intervals. The effective sample size of each parameter calculated in this inference was above 200. Tree samples in the MCMC were used to generate a maximum clade credibility tree using TreeAnnotator v.1.5.4 with a burn-in of 40000 states.

### Identification of Endemic Transmission Clusters

To identify viral transmission clusters, we performed three analyses; those matching all three approaches were recognized as monophyletic groups. The first approach was to evaluate the reliability of tree topology. We selected transmission cluster candidates from identical tree clusters with three different inference methods: NJ, maximum likelihood and maximum clade credibility tree in Bayesian MCMC. The second approach was a test of monophyly using the interior branch test in NJ tree and a posterior probability in Bayesian MCMC, in which significant clusters were determined as having ≥95% confidence probability for a target cluster. The third approach considered genetic divergence of the cluster against the whole sequence diversity. The distributions of all pairwise distances in the given phylogeny were calculated, and a specific sub-tree was then identified as a micro-clade if the median value of genetic distance between each pair of sub-tree members was lower than a threshold, determined as the 10^th^ percentile density (median diversity limit being 0.026) [42]. After a depth-first search of a rooted tree with all three approaches, small groups of viruses with clear evidence of common ancestry (posterior probability ≥0.95 in Baysian MCMC inference) were detected; we denoted these groups as “micro-clades”, as previously described for small groupings of circulating viral variants viruses [21]. Micro-clades were classified in two groups according to their origins; one is a cluster group having its ancestral virus from Japan (domestic micro-clade), and the other is a cluster group having its ancestral virus from foreign countries (international micro-clade). Micro-clades were then classified as transmission clusters with three or more cases, and cluster candidates as heterosexual, MSM or intravenous drug user (IVDU) pairs. The depth-first search of the clusters was computed by in-house scripts written in Perl 5. The tMRCA was estimated for each domestic micro-clade.

### Statistical Analysis

The distribution of IVDUs among micro-clades was tested by χ^2^ goodness of fit test to the Poisson distribution. All statistical analyses were calculated using R version 2.10.0.

## Results

### Major Risk Behavior among CRF01_AE Cases is Heterosexual Contact

We analyzed 297 protease-RT sequences from 243 CRF01_AE-infected cases, which corresponds to 6.7% of the collected study population of 3618 newly diagnosed patients from 2003 to 2010. The highest transmission category in both male and female CRF01_AE populations was high-risk heterosexual contact (Table 1). Among CRF01_AE patients, Japanese males were the majority (52.7%), but this predominance was significantly lower (two sided χ^2^ test, *p*<0.001) than in the overall population of HIV infected individuals in Japan (male: 93.2%, Japanese: 90.1%) [16]. The median ages for males, females and the total population were 45, 35 and 40 years, respectively. While patient nationality was not associated with heterosexual behavior (OR = 0.556; *p* = 0.183), MSMs and IVDUs were significantly more (OR = 15.444; *p*<0.001) and less frequent (OR = 0.086; *p* = 0.001) among Japanese patients, respectively (Table S3).

**Table 1:** Demographic Characteristics of CRF01_AE HIV-1-Infected Individuals in Japan (*N* = 243).

		Male		Female		Unknown	Total	
Characteristic		*n*	%	*n*	%	*n*	*n*	%
Nationality								
Japanese		128	52.7	36	14.8	1	165	67.9
Asian countries		19	7.8	31	12.8	0	50	20.6
China		1	0.4	3	1.2	0	4	1.6
Philippines		0	0.0	1	0.4	0	1	0.4
Vietnam		3	1.2	0	0.0	0	3	1.2
Malaysia		2	0.8	0	0.0	0	2	0.8
Indonesia		4	1.6	4	1.6	0	8	3.3
Thailand		5	2.1	19	7.8	0	24	9.9
Laos		1	0.4	1	0.4	0	2	0.8
Myanmar		3	1.2	3	1.2	0	6	2.5
South American countries		1	0.4	1	0.4	0	2	0.8
Brazil		0	0.0	1	0.4	0	1	0.4
Peru		1	0.4	0	0.0	0	1	0.4
Unspecified		4	1.6	2	0.8	0	6	2.5
Unknown		13	5.3	4	1.6	3	20	8.2
Transmission category								
High-risk heterosexual contact		104	42.8	62	25.5	0	166	68.3
Male-to-male sexual contact		37	15.2	NA	NA	0	37	15.2
Intravenous drug user		7	2.9	2	0.8	0	9	3.7
Unidentified		17	6.6	10	4.1	4	31	12.8
Area of clinics and facilities								
Region 1	(Hokkaido)	3	1.2	3	1.2	1	7	2.9
Region 2	(Kanto)	112	46.1	48	19.8	3	163	67.1
Region 3	(Koushinetsu)	4	1.6	4	1.6	0	8	3.3
Region 4	(Tokai)	26	10.7	14	5.7	0	40	16.5
Region 5	(Hokuriku)	1	0.4	1	0.4	0	2	0.8
Region 6	(Kinki)	15	6.2	2	0.8	0	17	7.0
Region 7	(Kyushu)	2	0.8	1	0.4	0	3	1.2
Region 8	(Okinawa)	2	0.8	1	0.4	0	3	1.2
Age, years								
20–29		33	13.6	21	8.6	0	54	22.2
30–39		36	14.8	26	10.7	0	62	25.5
40–49		49	20.2	11	4.5	0	60	24.7
50–59		26	10.7	8	3.3	0	34	14.0
60–69		20	8.2	7	2.9	0	27	11.1
>70		1	0.4	1	0.4	0	2	0.8
Unknown		0	0	0	0	4	4	1.6
Total		165	67.9	74	30.5	4	243	100

NA: not available.

### Phylogenetic Analysis Identifies 33 Micro-clades of CRF01_AE-Infected Patients in Japan

**Figure 2:**
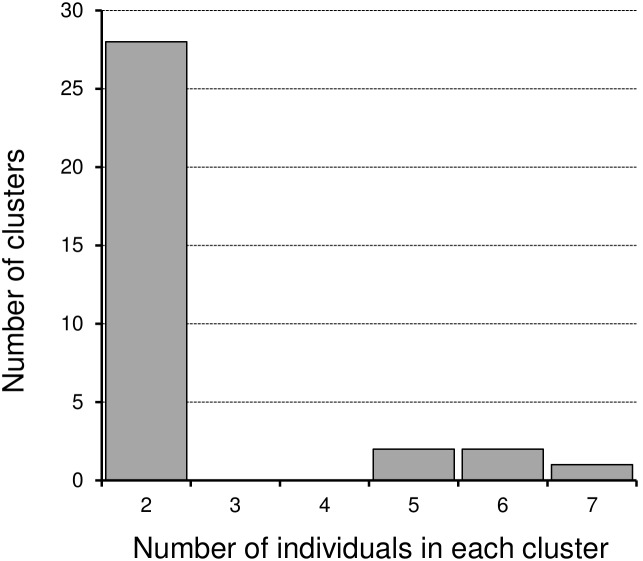
Histogram of CRF01_AE micro-clades by the number of individuals in each micro-clade. Twenty-eight micro-clades consisted of two patients and the remaining 5 micro-clades consisted of more than 4 individuals.

The estimated evolutionary diversity of the CRF01_AE protease-RT region is shown in Table S4. Coefficients of differentiation were low among the risk behaviors and collection areas. The mean evolutionary rate of the protease-RT region estimated by Bayesian MCMC inference was 1.07×10^−3^ substitutions per year (Table S5), consistent with previous estimates for the HIV genome [43]–[45]. The estimated mean coefficient of variation was 0.597, indicating substantial heterogeneity in the evolutionary rate in viral lineage (Table S5). The tree topology showed no association with any demographic parameters of patients. We identified 33 clusters that mainly spread in Japan, i.e., micro-clades MC01 to MC33 (Figure 1, Figure S2). Some of these micro-clades might have been due to intra-patient sequence variation, as suspected for MC27, 32 and 33. Other micro-clades seemed to come from inter-patient diversity because patients’ characteristics were clearly distinct from each other (Table 2). These micro-clades consisted of 76 patients (Table 2), corresponding to 31% of all CRF01_AE patients collected in our study. The distribution of cluster sizes is shown in Figure 2. Most micro-clades (n = 28, 85%) consisted of two patients, with only five micro-clades containing more than a pair.

**Table 2:** CRF01_AE domestic micro-clades (MCs) detected in the study population.

	tMRCA				Characteristics of individuals in micro-clades				
MC-ID	Median	95% HPD			Region	Age	Gender	Behavior	Nationality
Transmission Clusters									
MC01	Nov-‘96	Sep-‘93	–	Oct-‘01	Region 2	35	M	Hetero	Japan
					Region 2	42	F	Unknown	Japan
					Region 2	31	M	Hetero	Japan
					Region 3	44	F	Hetero	Thailand
					Region 2	37	F	Hetero	Japan
					Region 2	40	M	Hetero	Japan
MC02	Mar-‘00	Dec-‘96	–	Aug-‘02	Region 3	30	F	Hetero	Japan
					Region 2	29	M	Hetero	Vietnam
					Region 4	21	M	IVDU	Vietnam
					Reference	-	-	Unknown	Taiwan
					Reference	-	-	Unknown	Vietnam
MC03	Mar-‘01	Apr-‘98	–	Jun-‘03	Region 2	26	M	IVDU	Indonesia
					Region 2	47	M	Hetero	Japan
					Region 4	47	M	Hetero	Japan
					Region 2	32	M	IVDU	Indonesia
					Region 4	23	F	Hetero	Indonesia
					Region 4	26	F	Unknown	Indonesia
					Reference	-	-	Unknown	Korea
MC04	Feb-‘02	Sep-‘99	–	Jan-‘04	Region 2	21	F	IVDU	Japan
					Region 2	66	M	Unknown	Japan
					Region 2	45	F	Hetero	Japan
					Region 2	67	M	Hetero	Japan
					Region 2	60	M	MSM	Japan
					Region 2	63	M	Hetero	Japan
MC05	Jun-‘02	Feb-‘00	–	Apr-‘04	Region 2	28	F	Hetero	Japan
					Region 2	56	M	Hetero	Indonesia
					Region 4	24	F	IVDU	Indonesia
					Region 4	34	M	IVDU	Japan
					Region 4	35	M	IVDU	Indonesia
Heterosexual Male-Female Pairs									
MC06	Feb-‘03	Aug-‘00	–	Nov-‘04	Region 2	68	M	Hetero	Thailand
					Region 2	62	F	Hetero	Japan
MC07	May-‘03	Oct-‘01	–	Apr-‘04	Region 4	50	M	Hetero	Unknown
					Region 4	50	F	Hetero	Japan
MC08	Nov-‘03	Jan-‘02	–	Dec-‘04	Region 4	58	M	Hetero	Japan
					Region 4	51	F	Hetero	Japan
MC09	Jan-‘06	Oct-‘02	–	Jul-‘08	Region 2	40	M	Hetero	Myanmar
					Region 2	33	F	Hetero	Myanmar
MC10	Sep-‘06	Aug-‘03	–	Oct-‘08	Region 2	46	M	Hetero	Japan
					Region 2	29	F	Hetero	Japan
MC11	Apr-‘07	Jan-‘06	–	Dec-‘07	Region 4	66	M	Hetero	Japan
					Region 4	65	F	Hetero	Japan
MSM pairs									
MC12	Nov-‘01	Apr-‘00	–	Nov-‘02	Region 2	29	M	MSM	Japan
					Region 2	36	M	MSM	Japan
MC13	Aug-‘03	Jan-‘01	–	Jun-‘05	Region 6	43	M	MSM	Japan
					Region 6	56	M	MSM	Japan
MC14	Jan-‘07	Mar-‘05	–	May-‘08	Region 6	27	M	MSM	Japan
					Region 6	34	M	MSM	Japan
MC15	Feb-‘07	Mar-‘05	–	Apr-‘08	Region 2	46	M	MSM	Japan
					Region 2	40	M	MSM	Japan
Discordant couples with possible missing cases									
MC16	May-‘99	Jan-‘99	–	May-‘01	Region 2	28	M	Hetero	Japan
					Region 2	46	M	Hetero	Japan
MC17	Sep-‘99	May-‘96	–	Apr-‘02	Region 2	49	M	Hetero	Japan
					Region 2	28	M	MSM	Japan
MC18	Sep-‘00	Nov-‘96	–	Feb-‘03	Region 2	41	M	Hetero	Japan
					Region 2	52	M	Hetero	Japan
MC19	Oct-‘00	Nov-‘98	–	Jun-‘02	Region 4	52	M	Hetero	Japan
					Region 4	65	M	Hetero	Japan
MC20	Dec-‘00	May-‘98	–	Mar-‘03	Region 1	32	M	MSM	Japan
					Region 2	58	M	Hetero	Japan
MC21	Apr-‘03	Oct-‘99	–	Dec-‘05	Region 2	25	F	Hetero	Japan
					Region 2	39	M	MSM	Japan
MC22	Apr-‘05	Aug-‘03	–	Jun-‘06	Region 1	35	F	Hetero	Japan
					Region 2	59	F	Hetero	Japan
MC23	Mar-‘07	Sep-‘05	–	Jan-‘08	Region 2	44	F	Hetero	Japan
					Region 2	34	M	IVDU	non-Japanese
MC24	Sep-‘07	Dec-‘05	–	May-‘08	Region 6	26	M	MSM	Japan
					Region 2	25	M	Hetero	Japan
Insufficient information								^?^	?
MC25	Aug-‘04	Sep-‘02	–	Oct-‘05	Region 2	20s	M	Unknown	Unknown
					Region 2	28	M	MSM	Japan
MC26	Dec-‘05	Nov-‘02	–	Mar-‘08	Region 2	23	M	Unknown	Unknown
					Region 2	31	F	Hetero	Myanmar
MC27	Mar-‘07	Apr-‘06	–	Jun-‘07	Region 2	45	M	Hetero	Japan
					Region 4	44	M	Unknown	Japan
MC28	Apr-‘07	Jun-‘05	–	Apr-‘08	Region 2	35	F	Unknown	Unknown
					Region 2	29	F	Hetero	Thailand
MC29	May-‘07	Feb-‘05	–	Dec-‘08	Region 2	40s	M	Unknown	Unknown
					Region 2	73	M	Hetero	Japan
MC30	Jun-‘07	Jun-‘05	–	Oct-‘08	Region 2	47	M	Unknown	Unknown
					Region 2	35	M	Unknown	Unknown
MC31	Jul-‘08	Apr-‘07	–	Jan-‘09	Region 2	46	M	Hetero	Japan
					Region 4	39	M	Unknown	Japan
MC32	Oct-‘08	Oct-‘07	–	Jan-‘09	Region 2	24	M	Unknown	Unknown
					Region 2	25	M	MSM	Japan
MC33	Feb-‘09	Oct-‘07	–	Oct-‘09	Region 2	23	M	MSM	Japan
					Region 2	23	M	Unknown	Unknown

MC: micro-clade, HPD: highest posterior density, tMRCA: time of most recent common ancestor.

### Origin of the Transmission Clusters Introduced into Japan between 1996 and 2002

**Figure 3:**
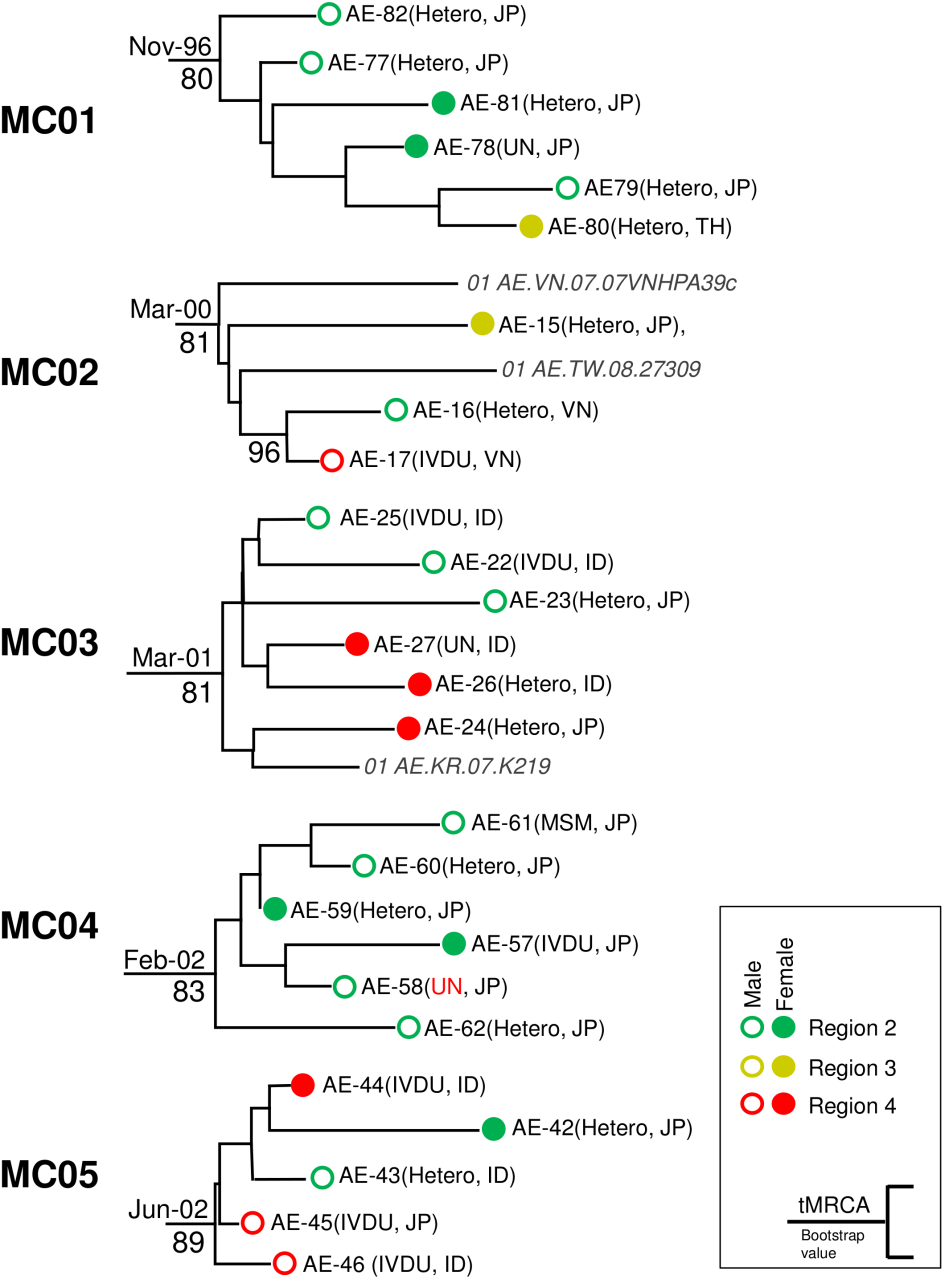
Detailed phylogenetic structure of five large micro-clades. Open and solid circles indicate male and female cases, respectively. Circle color indicates geographic origin of the samples, followed by risk behavior, and nationality, if known. Dates at the sub-tree’s root show the tMRCA of each micro-clade. Numbers below each node show bootstrap values above 80%. JP = Japanese, KR = Korean, VN = Vietnamese, ID = Indonesian, TH = Thai, TW = Taiwanese, UN = unknown, IDVU = intravenous drug user.

The median tMRCAs of micro-clades found in Japan and in foreign reference sequences are shown in Tables 2 and 3, respectively. The median tMRCA of CRF01_AE viruses in Japan was estimated using Bayesian MCMC inference as 1968 (95% HPD: 1975–1956), identical to that of the all CRF01_AE viruses measured in this study, including reference sequences. The earliest transmission cluster originating in Japan was MC01, with tMRCA of 1996. This cluster consisted of 6 individuals (3 males and 3 females), of whom 5 reported their risk behavior as heterosexual contact. Five individuals in MC01 were from Region 2, and one Thai female was detected in Region 3 (Figure 3). Four other transmission clusters (MC02-05) showed median tMRCAs between 2000 and 2002. As shown in Figure 3, these clusters comprised individuals from geographically wide areas of Japan, from eastern to central Japan (Regions 2, 3, 4 and 5; see also Figure S1). These clusters also included both genders and individuals of different nationalities, such as Japanese, Indonesian, Vietnamese, Taiwanese and South Korean, suggesting complex transmission networks in CRF01_AE. The most striking finding was that 7 of 9 IVDUs were significantly concentrated in these four large clusters (χ^2^ = 528.5; p<2.2×10^−16^).

**Table 3:** International micro-clades (IMCs) detected in the study population and outlier sequences.

	tMRCA				Characteristics of individuals in micro-clades					
IMC-ID	Median	95% HPD			Region	Age	Gender	Behavior		Nationality
IMC-1	Dec-‘94	Mar-‘93	–	Mar-‘96	Region 7	26	F	Hetero		Japan
					Region 4	24	M	Hetero		Japan
					Region 4	62	M	Hetero		Japan
					Region 2	35	M	MSM		Japan
					Region 2	54	M	Hetero		Japan
					Region 4	43	M	Unknown		Japan
					Region 2	66	M	Hetero		Japan
					Vietnam	35 ref seq (see Table S1)				
					China	7 ref seq (see Table S1)				
					France	2 ref seq (see Table S1)				
					Czech	2 ref seq (see Table S1)				
IMC-2	Oct-‘99	Dec-‘96	–	Dec-‘01	Region 4	32	F	Unknown		China
					China	27 ref seq (see Table S1)				
IMC-3	Feb-‘00	Aug-‘99	–	Dec-‘01	MC33	2 individuals in Japan (see Table 2)				
					China	12 ref seq (see Table S1)				
IMC-4	ND	ND		ND	China	4 ref seq (see Table S1)				
IMC-5	Aug-‘02	Jun-‘99	–	Feb-‘05	Region 2	30	M	MSM		Japan
					Philippines	3 ref seq (see Table S1)				
IMC-6	ND	ND		ND	China	23 ref seq (see Table S1)				
Sequence groups in CRF01_AE										
Japan AE	Nov-‘68	Sep-‘56	–	Apr-‘75	-	-				
Whole AE	Nov-‘68	Sep-‘56	–	Apr-‘75	-	-				

HPD: highest posterior density, tMRCA: time of most recent common ancestor, ND: not determined.

### Small CRF01_AE clusters frequently included MSM

In contrast to the large CRF01_AE clusters, which contained only 1 MSM/29 total individuals in clusters, the small CRF01_AE clusters frequently included individuals reporting MSM risk factors. The median tMRCA of small micro-clades ranged between 1999 and 2009 (Table 2). Among these micro-clades, 23 consisted of samples collected in the same region, whereas individuals in the remaining 5 micro-clades were widely distributed over mainland Japan (MC20, 22, 24, 27, and 31). Five micro-clades (MC06, 09, 23, 26, and 28) included at least one non-Japanese. The proportion of females in these small micro-clades was 23%, lower than that of the entire study population (30.5%, Table 1). Six micro-clades (MC06-11) consisted of heterosexual pairs that were unlikely to expand their transmission networks. The median tMRCAs of such closed micro-clades ranged from 2003 to 2007, and those of 4 micro-clades (MC12-15) consisting of MSM pairs ranged from 2001 to 2007. Other 26 small micro-clades had diverse demographic, and risk factor characteristics (Table 2). Taken together with the findings of the large clusters, these data highlight the complexity of CRF01_AE transmission in Japan.

### CRF01_AE Epidemic in Japan Occurred in at least Two Waves

**Figure 4:**
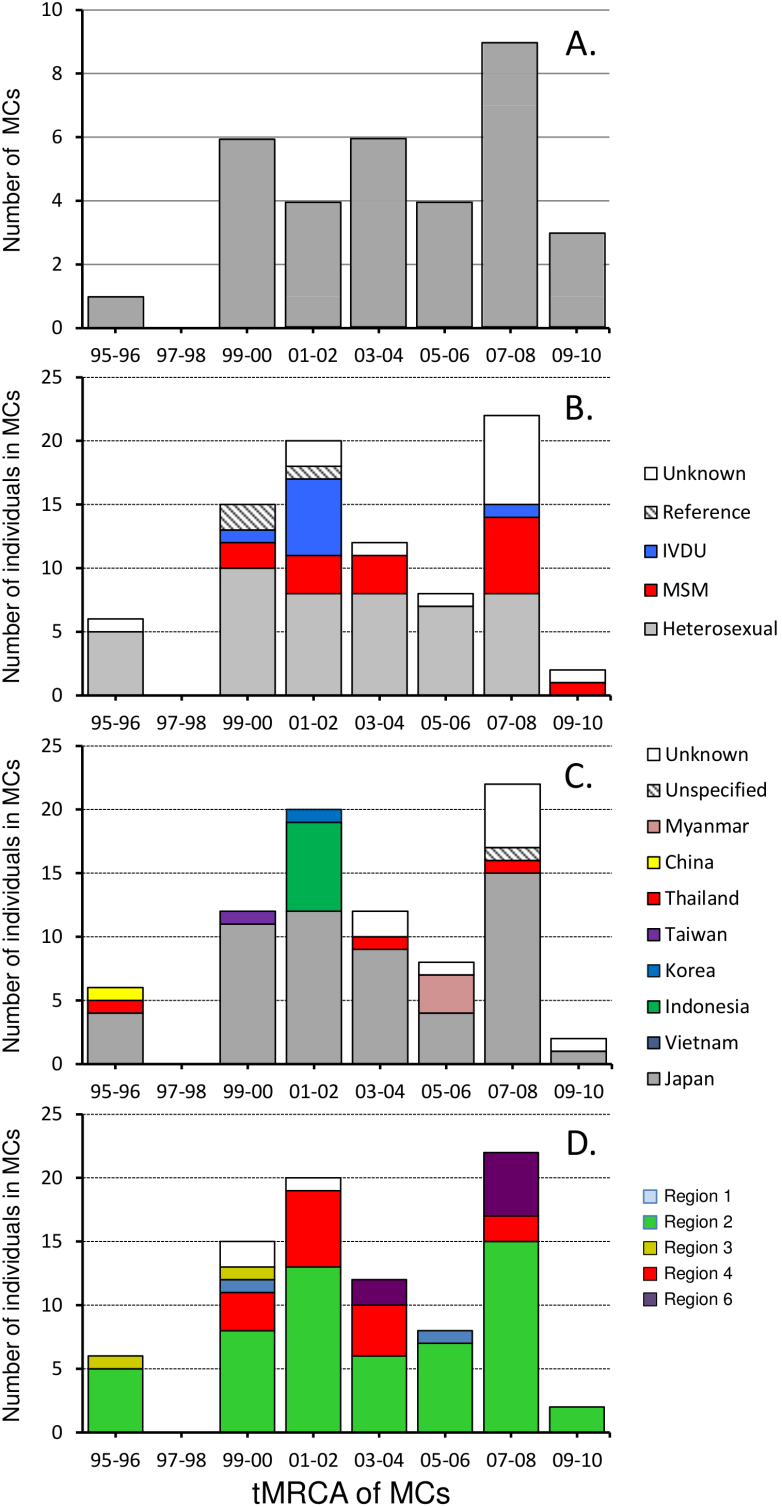
Dynamics of transmission clusters in Japan according to member individuals’ demographic characteristics. A) Number of individuals in micro-clades is shown versus micro-clade tMRCA (time of the most recent common ancestor). Bars in each panel are colored by B) risk behavior, C) nationality, and D) geographical region. MC =  micro-clade.

The distributions of numbers of individuals in micro-clades and their demographic parameters are graphed versus micro-clade tMRCA in Figure 4. Analysis of tMRCA revealed two distinct groups of CRF01_AE infected individuals: the first wave coming in the early 2000s and the second wave in 2007 to 2008 (Figure 4A). Before the first wave, the major risk factor in the clusters was heterosexual behavior, with a remarkable number of IVDUs around the first wave (Figure 4B). After the first wave, MSM increases gradually and reaches a peak at the second wave (Figure 4B). The clusters in the first wave included many Indonesians (Figure 4C). Thailand, where the CRF01_AE outbreak started, had few contributions to cluster formation in any years, and Japanese mainly contributed in the recent 4 years from 2006 to 2010 (Figure 4C). Individuals from Region 6 were found in the clusters only after 2003, showing that the infection seems to have spread from eastern Japan to the rest of the mainland around this year (Figure 4D). These patterns were not found in analyses of the relationship between demographics and collection year (Figure S3). These data suggest CRF01_AE was introduced at least twice, with one early wave occurring in the early 2000’s largely transmitted through heterosexual contact, and a second distinct wave of transmission occurring later that has been sustained by transmission through multiple risk factors, that includes a substantial contribution of MSM transmission.

### Some Individuals Infected with Viruses from the Transmission Clusters Spread in East and Southeast Asian Countries

Besides domestic micro-clades, 6 international micro-clades (IMC-1-6) were determined in reference CRF01_AE sequences from the Los Alamos HIV database (Figure 1, Table S1). Among these international micro-clades, 4 (IMCs 1-3 and 5) included sequences from our study (Table 3, Figures S4 and S5). Seven individuals from a wide range of regions in Japan were grouped into IMC-1, the largest international micro-clade with reference sequences collected in Vietnam and China. IMC-2, the second largest cluster consisting of 27 sequences from China, contained one Chinese female detected in Region 4. IMC-3 consisted of 12 sequences from China and included MC33, which included 1 or 2 Japanese males from Region 2. IMC-5, which contained reference sequences from the Philippines, included one MSM Japanese male from Region 2. Thus, our data clearly demonstrate ongoing international transmission of CRF01_AE, especially between China and Japan.

## Discussion

The results of this study reveal a process of multiple transmissions and a subsequent spreading pattern of CRF01_AE infection into Japan. Our sample represents 18.7% of the officially reported HIV-1-infected cases in Japan [16]. Although our results were obtained from the relatively short and genetically conserved *pol* sequence region, we collected the largest number of sequences for this region and we considered that this greater number would be more advantageous for our study. The age distribution of CRF01_AE is shifted more toward older populations compared to subtype B cases. However, as non-B subtype HIV-1-infected individuals tend to visit a clinic when they have not recently seroconverted (>155 days) [16], the estimated age at infection may be similar to that for subtype B HIV-infected cases. Unlike subtype B-infected cases, among which MSM is the major risk factor, more than half of the CRF01_AE-infections in Japan were estimated to be transmitted through heterosexual contact.

Using composite phylogenetic analysis, we determined at least 30 clusters of CRF01_AE-infected individuals in Japan; we noted 6 groups had international members, as they contained both our surveillance data and reference sequences from other Asian countries. Among 243 patient samples collected in the study, 76 were from patients involved in these micro-clades, demonstrating transmission networks of the CRF01_AE infection in Japan. The remaining 167 samples failed to be identified in transmission clusters; the CRF01_AE epidemic is quite large, and it is possible that sequences without identifiable partners is due to the low coverage rate of our surveillance or that they were newly imported from neighboring countries.

An original outbreak of CRF01_AE among the high-risk heterosexual population was first reported in Thailand in the late 1980s [4]–[6], then disseminated to neighboring countries [8]–[12]. Soon after the outbreak in Thailand, the first extensive colonization of the virus was estimated in Japan. Indeed, our phylodynamic analysis suggests that the primary domestic transmission cluster of CRF01_AE was formed in the 1990s. This possibility was confirmed from IMC-1, which includes 7 domestic sequences and “cluster 3″ containing sequences from North Vietnam and a neighboring Chinese province described by Liao et al. [46], suggesting simultaneous disseminations to these countries, including Japan (Figure S5). Our tMRCA analysis demonstrated that some transmission clusters independently spread in East Asia from around 2000 (Table 3). Among these, IMC-1 was the same as the cluster found in IVDU populations in Northern Vietnam and Southeastern China [46], [47]. The estimated median tMRCA of IMC-1 was similar to that of previous studies [46], [47], and our subjects from regions 2 and 4 were scattered within the cluster, suggesting repeated transmission events of CRF01_AE from these regions to Japan around 2000. In turn, IMC-3 was the same as a cluster recently found in a northeastern Chinese MSM population (named “cluster 1″ [48]–[50] or CN.MSM-01-01 [51]) and included the domestic micro-clade, MC33.e constructed a maximum likelihood tree using our subjects with 4 CN.MSM-01-01 sequences derived from Japan with the same substitution model and confirmed that members of MC33 were involved in a Japanese sub-cluster of CN.MSM-01-01 (Figure S6). Therefore, one can observe that MC33-related viruses may have contributed an outbreak in an MSM community in metropolitan areas of Japan. Additionally, domestic clusters MC02 and MC03 included reference sequences originating from Taiwan and Vietnam, and South Korea, respectively (Figure 3). Thus our data clearly suggest that transmission networks of CRF01_AE have developed between Japan and other Asian countries from the first colonization wave. The CRF01_AE epidemic scenario would be that CRF01_AE was initially imported to Japan in the 1990s from neighboring Asian countries especially by IVDU behavior. Then, Japanese variants may have influenced epidemics among MSM in East Asian countries, as suggested by Kondo et al [51], by exporting cases in the middle of the 2000s. Among such “connected” countries in Asia, generation of recombinant forms between CRF01_AE and other subtypes has been a recent concern [7], [51].

After the primary dissemination to Japan, our tMRCA distribution data strongly suggest that CRF01_AE invaded Japan in two substantial waves, one in 2000 and the other in 2007. Our finding is based on the ability to detect common ancestors among circulating viral variants. We could not assign common ancestors to all of our sequences, and the CRF01_AE epidemic is substantial and genetically diverse. As a result, our sampling, as well as sampling in other countries, may not be sufficient to detect additional, ongoing introductions of CRF01_AE. A serious concern drawn from our findings is the role of IVDU in the CRF01_AE transmission network. The concentration of IVDUs observed in large micro-clades (e.g., MC05) indicates a suspected linkage of high-risk sexual communities and drug addicts, including international partners (MC05) As stigma often limits standard risk assessment for HIV, the phylodynamic approach described here offers an effective method to track ongoing epidemics within suspected IVDU communities, with the results of our analysis indeed clarifying critical contributions of IVDU to the CRF01_AE outbreak in Japan. These results aid in calling attention to the need to focus resources on interventions designed to specifically limit spread among specific risk groups to curtail CRF01_AE transmissions through the IVDU route.

## Supporting Information

Figure S1 **Geographic location of HIV-1 sample collection regions in Japan.** Regions of sample collection are designated by the same colors used to indicate sample origin in other figures.
(PDF)

Figure S2 **Distance-based neighbor joining phylogeny of the protease-RT region of CRF01_AE HIV-1 in Japan.** Numbers on each branch show the results of interior branch testing, where probabilities >95%. The sequences obtained in our surveillance network are designated by circles in different colors according to the region of sample collection. Reference sequences from the Los Alamos database are designated by black triangles. Micro-clades and significant clusters are annotated by red branches with brackets on the right of the tree. Scale bar at the bottom shows the number of nucleotide substitutions per site.
(PDF)

Figure S3 **Distribution of sample collection time of CRF01_AE HIV-1-infected individuals in Japan.** The cumulative numbers of CRF01_AE HIV-1-infected individuals are shown by year of sample collection. Bars in each panel are colored by individuals’ A) gender and risk behavior, B) nationality, and C) geographical region.
(PDF)

Figure S4 **Partial chronological phylogenetic tree of IMC-1.** An international micro-clade including 7 sequences from our study population and a cluster that spread mainly in Vietnam [46] extracted from the Bayesian MCMC phylogeny is shown. The 7 sequences are designated by symbols according to their gender and the region of sample collection. JP = Japanese; UN = unknown.
(PDF)

Figure S5 **Partial chronological phylogenetic tree of IMC-3.** An international micro-clade composed of CRF01_AE sequences found in China extracted from the Bayesian MCMC phylogeny is shown. This cluster included MC15. Sequences are designated by symbols according to their gender and the region of sample collection. JP = Japanese; UN = unknown.
(PDF)

Figure S6 **Maximum likelihood phylogenetic tree of the large Chinese cluster CN.MSM.01-01 with MC33.** Protease-RT sequences belonging to CN.MSM.01-01 [51] were selected from the Los Alamos database and aligned with our study subjects and outlier sequences. Maximum likelihood phylogeny was inferred from the alignment as described in Materials and Methods. A partial tree including CN.MSM.01-01 is represented. Numbers below branches indicate bootstrap probability. Japanese sequences in CN.MSM.01-01 are underlined. Our sequences are designated by symbols according to their gender and the region of sample collection. JP = Japanese; UN = unknown.
(PDF)

Table S1 **CRF01_AE outlier sequences from the Los Alamos HIV database.**
(PDF)

Table S2 **Bayesian factor analysis of molecular clock models compared for constant demographic size.**
(PDF)

Table S3 **Independence test of risk behaviors and nationalities in CRF01_AE-infected patients in Japan.**
(PDF)

Table S4 **Estimates of the mean evolutionary diversity for categories of CRF01_AE sequences.**
(PDF)

Table S5 **Evolutionary parameters obtained in Bayesian MCMC inference with constant size and lognormal relaxed.**
(PDF)
