# Supplementary figures and images for: Phylodynamic Analysis Reveals CRF01_AE Dissemination between Japan and Neighboring Asian Countries and the Role of Intravenous Drug Use in Transmission
Source: PLoS One. 2014 Jul 15;9(7):e102633. doi: 10.1371/journal.pone.0102633 (PMC4099140; doi:10.1371/journal.pone.0102633)

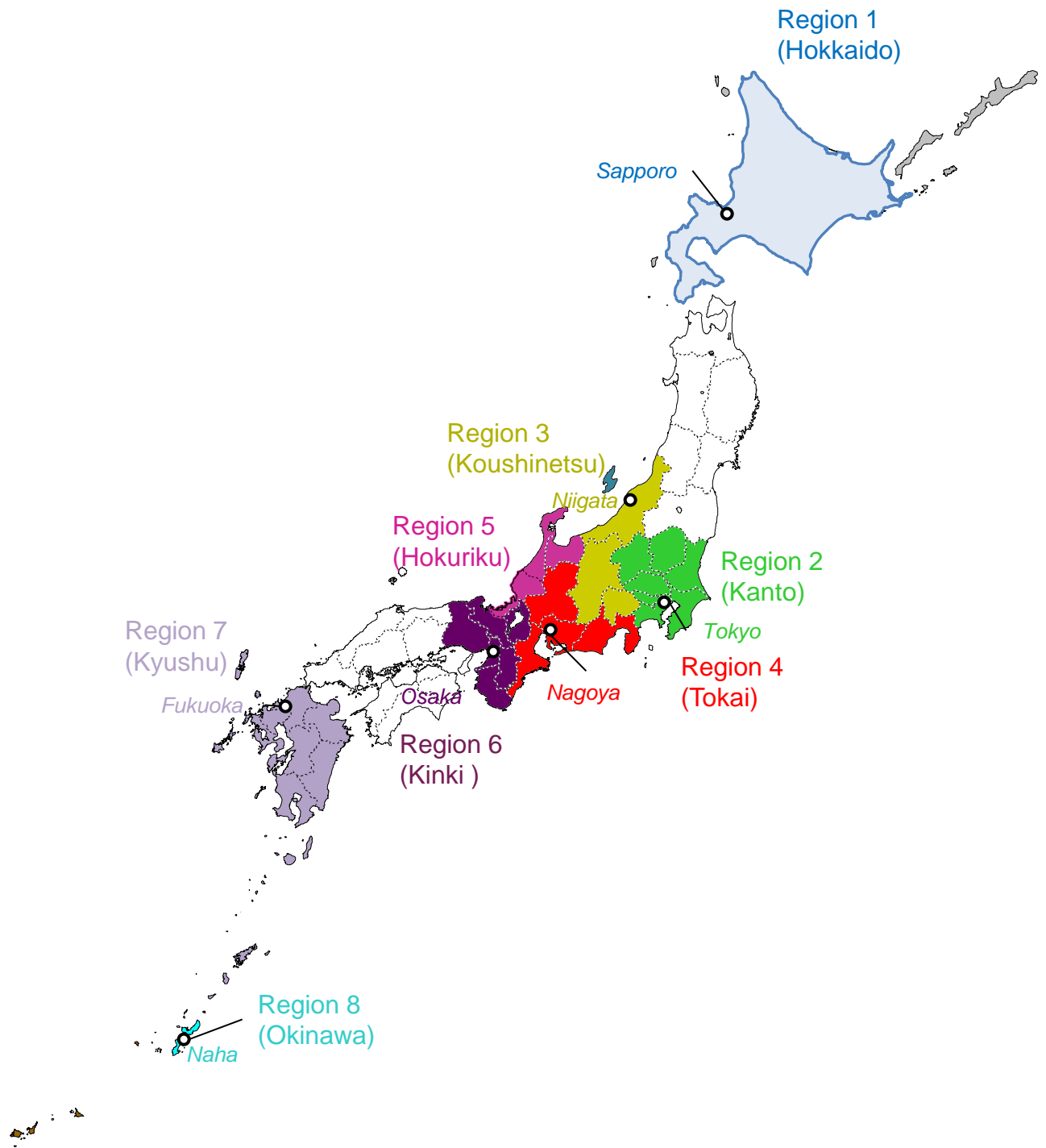

Supplement: Figure S1 — Geographic location of HIV-1 sample collection regions in Japan. Regions of sample collection are designated by the same colors used to indicate sample origin in other figures. (PDF) [file pone.0102633.s001.pdf]

- Region 1
- Region 2
- Region 3
- Region 4
- Region 5
- Region 6
- Region 7
- Region 8
- ◄ Foreign reference

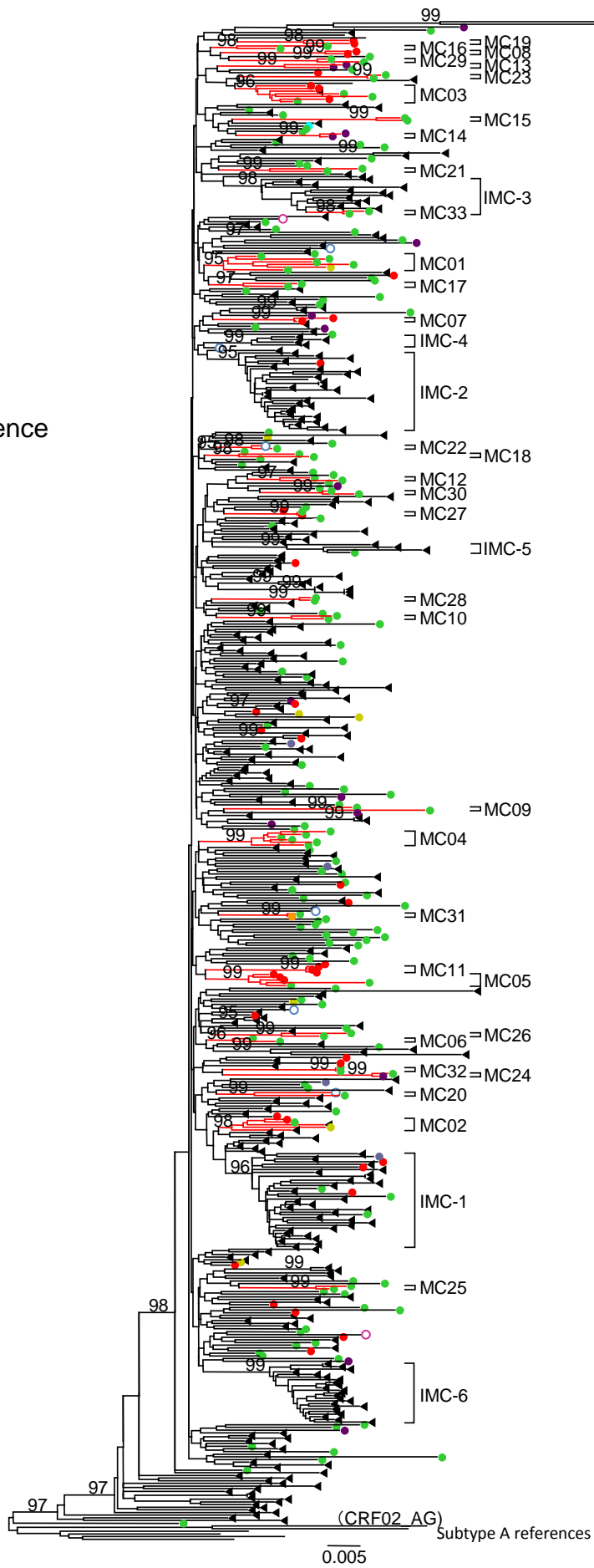

Supplement: Figure S2 — Distance-based neighbor joining phylogeny of the protease-RT region of CRF01_AE HIV-1 in Japan. Numbers on each branch show the results of interior branch testing, where probabilities >95%. The sequences obtained in our surveillance network are designated by circles in different colors according to the region of sample collection. Reference sequences from the Los Alamos database are designated by black triangles. Micro-clades and significant clusters are annotated by red branches with brackets on the right of the tree. Scale bar at the bottom shows the number of nucleotide substitutions per site. (PDF) [file pone.0102633.s002.pdf]

Number of CRF01\_AE-infected individuals in Japan

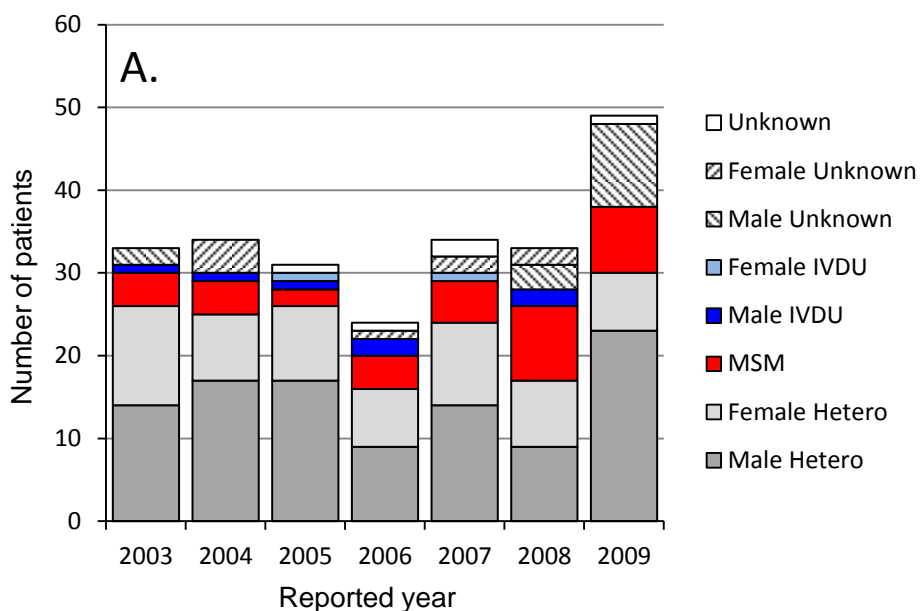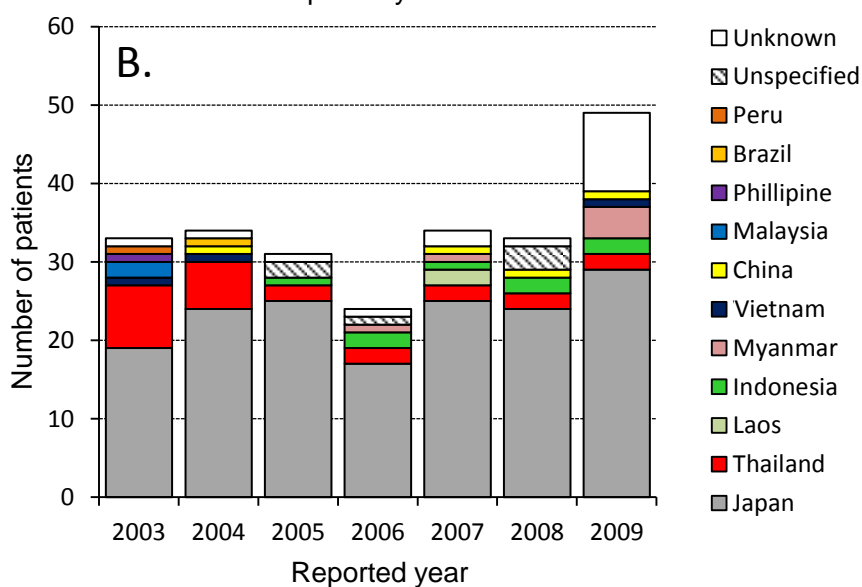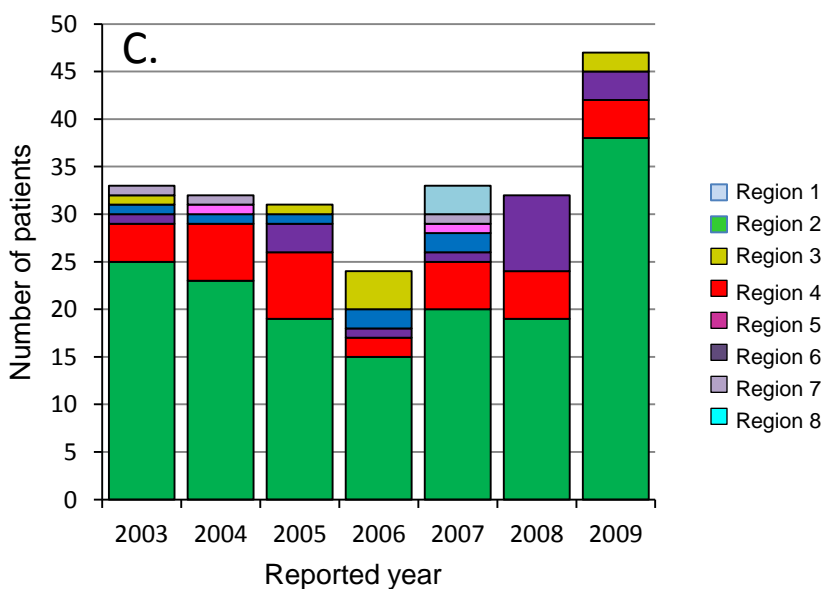

Supplement: Figure S3 — Distribution of sample collection time of CRF01_AE HIV-1-infected individuals in Japan. The cumulative numbers of CRF01_AE HIV-1-infected individuals are shown by year of sample collection. Bars in each panel are colored by individuals’ A) gender and risk behavior, B) nationality, and C) geographical region. (PDF) [file pone.0102633.s003.pdf]

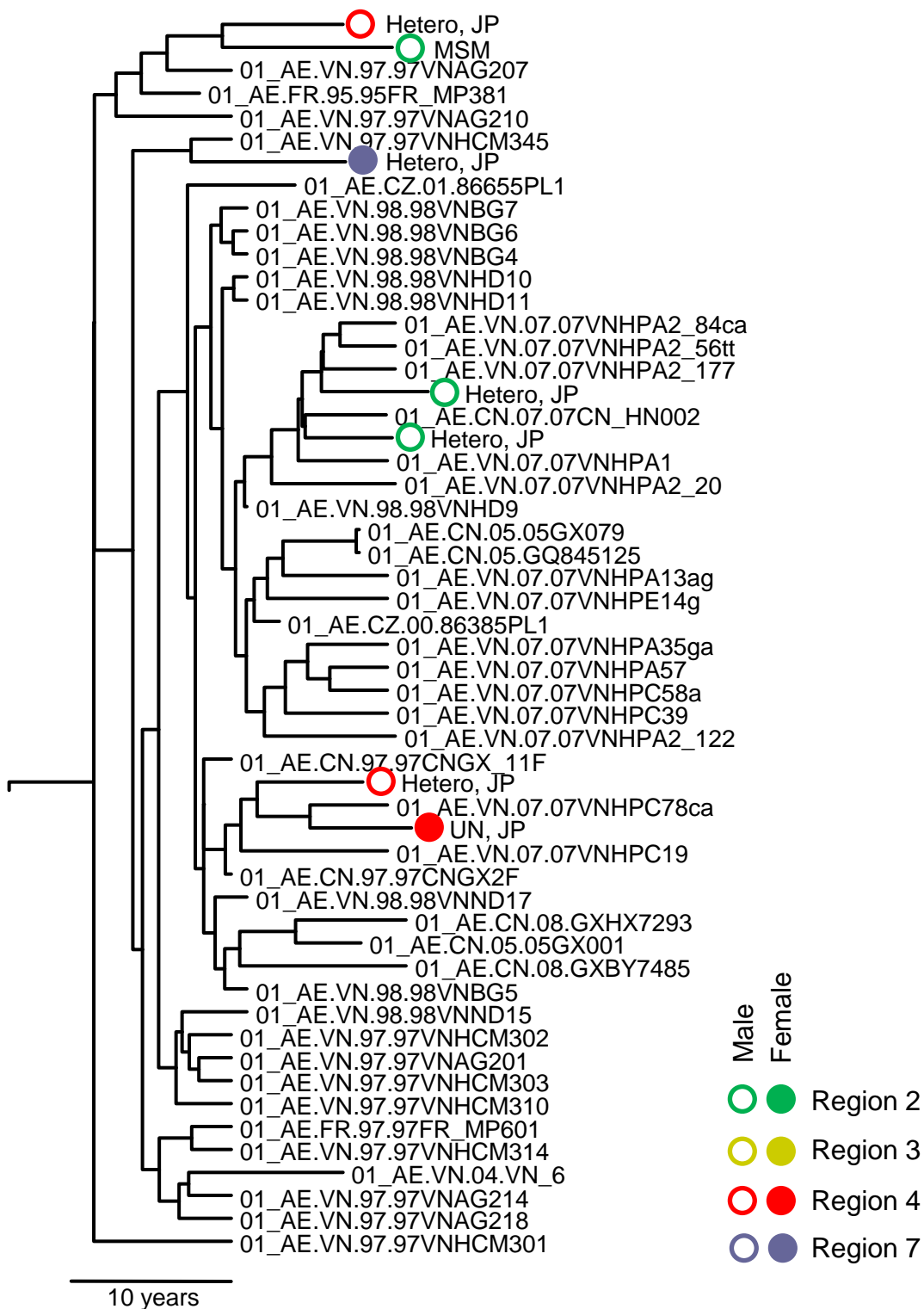

Supplement: Figure S4 — Partial chronological phylogenetic tree of IMC-1. An international micro-clade including 7 sequences from our study population and a cluster that spread mainly in Vietnam [46] extracted from the Bayesian MCMC phylogeny is shown. The 7 sequences are designated by symbols according to their gender and the region of sample collection. JP = Japanese; UN = unknown. (PDF) [file pone.0102633.s004.pdf]

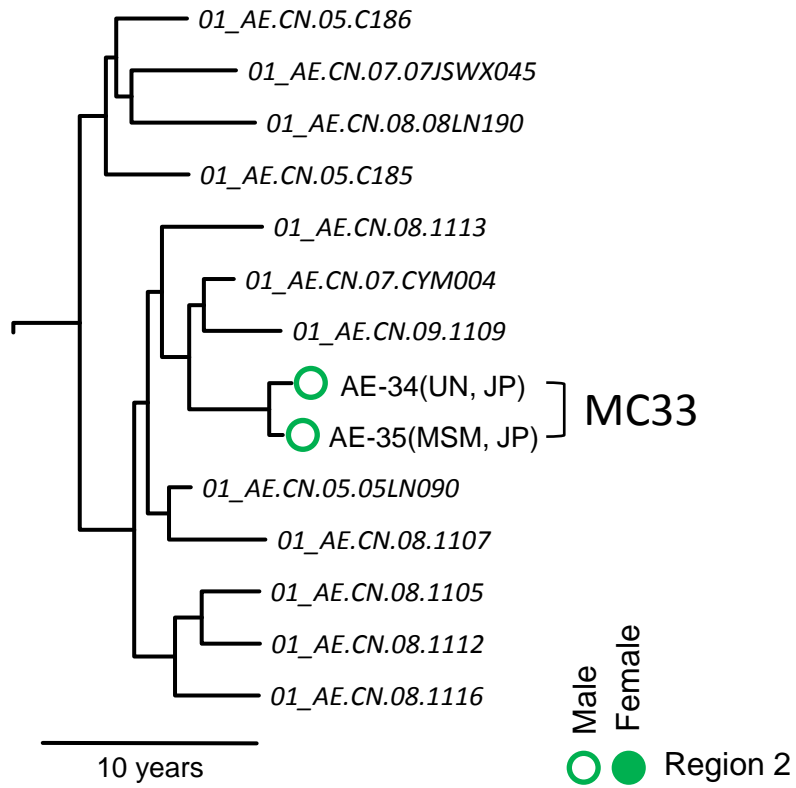

Supplement: Figure S5 — Partial chronological phylogenetic tree of IMC-3. An international micro-clade composed of CRF01_AE sequences found in China extracted from the Bayesian MCMC phylogeny is shown. This cluster included MC15. Sequences are designated by symbols according to their gender and the region of sample collection. JP = Japanese; UN = unknown. (PDF) [file pone.0102633.s005.pdf]

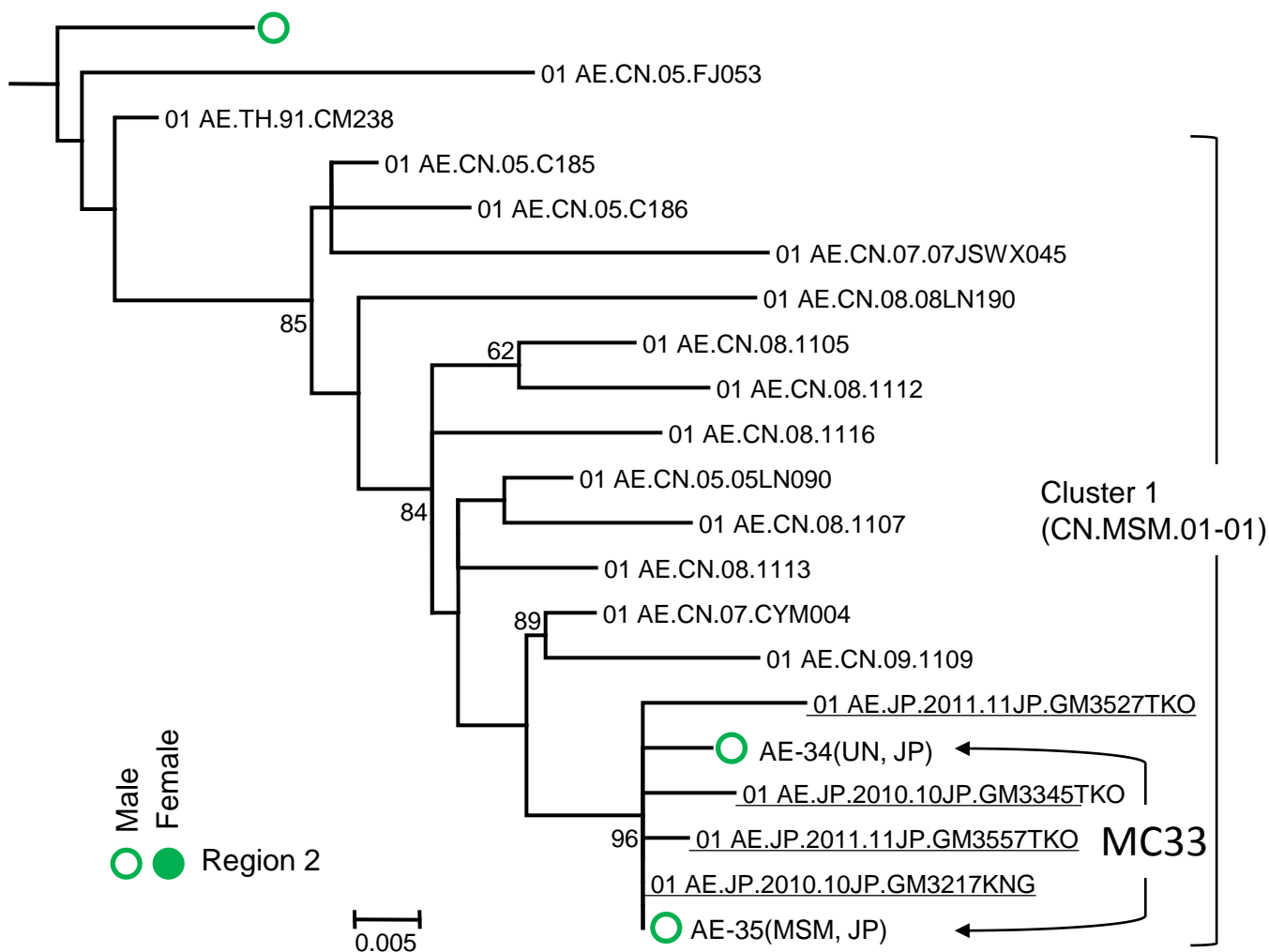

Supplement: Figure S6 — Maximum likelihood phylogenetic tree of the large Chinese cluster CN.MSM.01-01 with MC33. Protease-RT sequences belonging to CN.MSM.01-01 [51] were selected from the Los Alamos database and aligned with our study subjects and outlier sequences. Maximum likelihood phylogeny was inferred from the alignment as described in Materials and Methods. A partial tree including CN.MSM.01-01 is represented. Numbers below branches indicate bootstrap probability. Japanese sequences in CN.MSM.01-01 are underlined. Our sequences are designated by symbols according to their gender and the region of sample collection. JP = Japanese; UN = unknown. (PDF) [file pone.0102633.s006.pdf]
