# Supplementary material for: Phylodynamic Analysis Reveals CRF01_AE Dissemination between Japan and Neighboring Asian Countries and the Role of Intravenous Drug Use in Transmission
Source: PLoS One. 2014 Jul 15;9(7):e102633. doi: 10.1371/journal.pone.0102633 (PMC4099140; doi:10.1371/journal.pone.0102633)
Supplement: Table S1 — CRF01_AE outlier sequences from the Los Alamos HIV database. (PDF) [file pone.0102633.s007.pdf]

Table S1. CRF01_AE outlier sequences from the Los Alamos HIV database

Sequence Name	Accession	Country	Year	Cluster ID
01_AE.AF.07.569M	GQ477441	Afghanistan	2007	
01_AE.AT.04.gtHKAHTyyVbQPPL	GQ39883	Austria	2004	
01_AE.BE.02.AR02_480	AJ634694	Belgium	2002	
01_AE.BE.03.128570520380785	EU248299	Belgium	2003	
01_AE.BE.03.2kt4QRmIN7aV8iv	EU248334	Belgium	2003	
01_AE.BE.03.2znWScaURJNunLH	EU248336	Belgium	2003	
01_AE.BE.03.412883916880448	EU248366	Belgium	2003	
01_AE.BE.03.447237238710385	EU248377	Belgium	2003	
01_AE.CM.01.VP_ES_35	GU207116	Cameroon	2001	
01_AE.CM.01.VP_NW_12	GU207125	Cameroon	2001	
01_AE.CM.01.VP_OU_35-01	GU207108	Cameroon	2001	
01_AE.CM.03.CK22_35-01	AM279456	Cameroon	2003	
01_AE.CM.09.CIRCB-11004-01	JQ796132	Cameroon	2009	
01_AE.CF.90.90CF11697	AF197340	Central African Rep.	1990	
01_AE.CF.90.90CF402	AF197342	Central African Rep.	1990	
01_AE.CF.90.90CF4071	AF197341	Central African Rep.	1990	
01_AE.CF.90.90CR402	U51188	Central African Rep.	1990	
01_AE.TD.06.HG_0344	GU191660	Chad	2006	
01_AE.CN.96.96CNKM003	AB213669	China	1996	
01_AE.CN.97.97CNGX_11F	AY008718	China	1997	IMC-1
01_AE.CN.97.97CNGX2F	AY008714	China	1997	IMC-1
01_AE.CN.02.02CNLN27	EF122518	China	2002	
01_AE.CN.03.03CNLN47	EF122530	China	2003	
01_AE.CN.04.04CNLN61	EF122534	China	2004	IMC-6
01_AE.CN.05.05GX001	GU564221	China	2005	IMC-1
01_AE.CN.05.05GX002	GU564222	China	2005	IMC-2
01_AE.CN.05.05GX012	GU564223	China	2005	IMC-2
01_AE.CN.05.05GX013	GU564224	China	2005	IMC-2
01_AE.CN.05.05GX014	GU564225	China	2005	IMC-2
01_AE.CN.05.05GX034	GQ845124	China	2005	IMC-2
01_AE.CN.05.05GX079	GQ845125	China	2005	IMC-1
01_AE.CN.05.05GX128	GQ845126	China	2005	IMC-2
01_AE.CN.05.05LN087	FJ531405	China	2005	IMC-6
01_AE.CN.05.05LN090	FJ531406	China	2005	IMC-3

01_AE.CN.05.05LN092	FJ531407	China	2005	IMC-6
01_AE.CN.05.C073	GQ290706	China	2005	
01_AE.CN.05.C074	GQ290707	China	2005	IMC-6
01_AE.CN.05.C102	GQ290710	China	2005	
01_AE.CN.05.C185	GQ290722	China	2005	IMC-2
01_AE.CN.05.C186	GQ290723	China	2005	IMC-3
01_AE.CN.05.FJ051	DQ859178	China	2005	IMC-3
01_AE.CN.05.Fj052	EF036528	China	2005	
01_AE.CN.05.FJ053	DQ859179	China	2005	
01_AE.CN.05.Fj055	EF036527	China	2005	
01_AE.CN.05.Fj056	EF036529	China	2005	IMC-2
01_AE.CN.05.Fj057	EF036530	China	2005	
01_AE.CN.05.Fj065	EF036534	China	2005	
01_AE.CN.05.Fj066	EF036535	China	2005	
01_AE.CN.06.06CN_YUN01_01	AM886178	China	2006	
01_AE.CN.06.06CNgh177	EF394230	China	2006	
01_AE.CN.06.06LN103	FJ531408	China	2006	IMC-6
01_AE.CN.06.06LN105	FJ531409	China	2006	IMC-6
01_AE.CN.06.06LN109	FJ531411	China	2006	IMC-6
01_AE.CN.06.06LN111	FJ531412	China	2006	IMC-6
01_AE.CN.06.06LN112	FJ531413	China	2006	IMC-6
01_AE.CN.06.FJ054	DQ859180	China	2006	
01_AE.CN.06.Fj061	EF036536	China	2006	
01_AE.CN.06.Fj062	EF036531	China	2006	
01_AE.CN.06.Fj063	EF036532	China	2006	
01_AE.CN.06.Fj064	EF036533	China	2006	
01_AE.CN.07.07CN_HN002	FM251948	China	2007	IMC-1
01_AE.CN.07.07CN_HN005	FM251951	China	2007	IMC-2
01_AE.CN.07.07CN_HN006	FM251952	China	2007	IMC-2
01_AE.CN.07.07CN_HN008	FM251954	China	2007	
01_AE.CN.07.07CN_HN010	FM251956	China	2007	IMC-2
01_AE.CN.07.07CN_HN018	FM251962	China	2007	IMC-2
01_AE.CN.07.07CN_HN019	FM251963	China	2007	IMC-2
01_AE.CN.07.07CN_HN020	FM251964	China	2007	IMC-2
01_AE.CN.07.07CN_HN024	FM251968	China	2007	
01_AE.CN.07.07CN_HN025	FM251969	China	2007	

01_AE.CN.07.07CN_HN036	FM251978	China	2007	IMC-2
01_AE.CN.07.07CN_HN037	FM251979	China	2007	IMC-2
01_AE.CN.07.07CN_HN038	FM251980	China	2007	IMC-2
01_AE.CN.07.07CN_HN039	FM251981	China	2007	IMC-2
01_AE.CN.07.07CN_HN040	FM251982	China	2007	IMC-2
01_AE.CN.07.07JSWX045	FJ441290	China	2007	IMC-3
01_AE.CN.07.07LN128	FJ531424	China	2007	IMC-6
01_AE.CN.07.07LN132	FJ531425	China	2007	IMC-6
01_AE.CN.07.07LN138	FJ531428	China	2007	IMC-6
01_AE.CN.07.07LN153	FJ531429	China	2007	IMC-6
01_AE.CN.07.07LN154	FJ531430	China	2007	IMC-6
01_AE.CN.07.07SY023	FJ531442	China	2007	IMC-6
01_AE.CN.07.07SY041	FJ531443	China	2007	IMC-6
01_AE.CN.07.07SY123	FJ531444	China	2007	IMC-6
01_AE.CN.07.07SY147	FJ531445	China	2007	IMC-6
01_AE.CN.07.07SY40	FJ531459	China	2007	IMC-6
01_AE.CN.07.CYM004	EU921955	China	2007	IMC-3
01_AE.CN.08.08LN190	FJ531437	China	2008	IMC-3
01_AE.CN.08.08LN191	FJ531438	China	2008	
01_AE.CN.08.08LN194	FJ531439	China	2008	IMC-6
01_AE.CN.08.08LN197	FJ531440	China	2008	IMC-6
01_AE.CN.08.08LN203	FJ531441	China	2008	IMC-6
01_AE.CN.08.1105	HQ215569	China	2008	IMC-3
01_AE.CN.08.1107	HQ215581	China	2008	IMC-3
01_AE.CN.08.1112	HQ215584	China	2008	IMC-3
01_AE.CN.08.1113	HQ215585	China	2008	IMC-3
01_AE.CN.08.1116	HQ215586	China	2008	IMC-3
01_AE.CN.08.GXBY5817	HQ588239	China	2008	IMC-2
01_AE.CN.08.GXBY5848	HQ588250	China	2008	IMC-2
01_AE.CN.08.GXBY7485	HQ588258	China	2008	IMC-1
01_AE.CN.08.GXHX1605	HQ588245	China	2008	
01_AE.CN.08.GXHX1922	HQ588248	China	2008	IMC-2
01_AE.CN.08.GXHX7293	HQ588269	China	2008	IMC-1
01_AE.CN.08.GXNN2983	HQ588240	China	2008	IMC-2
01_AE.CN.08.GXNN5820	HQ588233	China	2008	IMC-2
01_AE.CN.08.GXNN5851	HQ588234	China	2008	IMC-2

01_AE.CN.08.GZ0801	FJ752409	China	2008	IMC-4
01_AE.CN.08.GZ0802	FJ752410	China	2008	IMC-4
01_AE.CN.08.GZ0803	FJ752411	China	2008	IMC-4
01_AE.CN.08.GZ0804	FJ752412	China	2008	IMC-4
01_AE.CN.08.LZ1007	HQ588180	China	2008	IMC-2
01_AE.CN.08.LZ1009	HQ588181	China	2008	
01_AE.CN.08.LZ2055	HQ588197	China	2008	IMC-2
01_AE.CN.08.LZ2056	HQ588198	China	2008	
01_AE.CN.09.1109	HQ215555	China	2009	IMC-3
01_AE.CN.09.1119	HQ215553	China	2009	IMC-6
01_AE.CZ.00.52293PL11	AY694224	Czech Republic	2000	
01_AE.CZ.00.52293PL12	AY694311	Czech Republic	2000	
01_AE.CZ.00.73087PL12	AY694246	Czech Republic	2000	
01_AE.CZ.00.76400PL7	AY694242	Czech Republic	2000	
01_AE.CZ.00.84746PL1	AY694268	Czech Republic	2000	
01_AE.CZ.00.86385PL1	AY694313	Czech Republic	2000	IMC-1
01_AE.CZ.01.86655PL1	AY694329	Czech Republic	2001	IMC-1
01_AE.CZ.01.86677PL1	AY694322	Czech Republic	2001	
01_AE.CD.02.02CD_KP180	AM040989	Dem. Rep. Of Congo	2002	
01_AE.CD.02.02CD_KTB038	AM041036	Dem. Rep. Of Congo	2002	
01_AE.CD.07.CRF01_07CD3458	FR666647	Dem. Rep. Of Congo	2007	
01_AE.DK.96.3_bPI_pre	AM933273	Denmark	1996	
01_AE.DK.00.215	AJ419445	Denmark	2000	
01_AE.DK.00.236	AJ419459	Denmark	2000	
01_AE.DK.00.284	AJ419493	Denmark	2000	
01_AE.DK.00.309	AJ419514	Denmark	2000	
01_AE.DK.03.hSpq6jLKX6B1lXZ	GQ398840	Denmark	2003	
01_AE.FI.05.mMLnxtzAE6mUAHd	GQ398890	Finland	2004	
01_AE.FR.95.95FR_MP381	AJ287043	France	1995	IMC-1
01_AE.FR.97.97FR_MP601	AJ287053	France	1997	IMC-1
01_AE.GA.00.00GAB_16M	AJ313417	Gabon	2000	
01_AE.DE.98.982414	AF347337	Germany	1998	
01_AE.DE.04.F5vimU1WoQKgBwz1	GQ400613	Germany	2004	
01_AE.HK.04.HK001	DQ234790	Hong Kong	2004	
01_AE.ID.93.ID12	AF447824	Indonesia	1993	
01_AE.ID.93.ID17	AB485652	Indonesia	1993	

01_AE.IT.00.DOTA520514	AY352445	Italy	2000	
01_AE.IT.00.s03_937	AY352445	Italy	2000	
01_AE.IT.00.s55_1004	AY368987	Italy	2000	
01_AE.IT.00.Tch74	AY359515	Italy	2000	
01_AE.IT.00.Yan74	AY366537	Italy	2000	
01_AE.IT.08.CV1193_88_08	GU969548	Italy	2008	
01_AE.MM.09.09mIDU058	JN223151	Myanmar	2009	
01_AE.NL.03.rsTwOeemdWctDaZ	GQ398952	Netherlands	2003	
01_AE.NO.05.kVFYjAXe2ni9XZv	GQ398868	Norway	2005	
01_AE.PK.08.M_I_380	JN620508	Pakistan	2008	
01_AE.PH.08.08R_01_359	AB587102	Philippines	2008	IMC-5
01_AE.PH.09.09MM_09_1840	AB587100	Philippines	2009	IMC-5
01_AE.PH.10.09MM_09_8567	AB587097	Philippines	2010	IMC-5
01_AE.PH.10.10MC_01_52	AB587106	Philippines	2010	
01_AE.PL.03.498_1	GU906862	Poland	2003	
01_AE.PL.03.761862264994976	GQ400086	Poland	2003	
01_AE.PL.09.335_28	GU906859	Poland	2009	
01_AE.RU.99.RU99029	EU345862	Russian Federation	1999	
01_AE.SN.98.98SN_MP1152	AJ287011	Senegal	1998	
01_AE.CS.03.249354600236553	GQ399221	Serbia	2003	
01_AE.SG.03.BAA148356	AY870142	Singapore	2003	
01_AE.SG.03.BAA148357	AY870143	Singapore	2003	
01_AE.SG.03.BAA148358	AY870144	Singapore	2003	
01_AE.SG.03.BAA148360	AY870145	Singapore	2003	
01_AE.SG.03.BAA148361	AY870146	Singapore	2003	
01_AE.SG.03.S07057	EU715231	Singapore	2003	
01_AE.SG.04.S07007	EU715184	Singapore	2004	
01_AE.SG.06.S07003	EU715177	Singapore	2006	
01_AE.SG.07.S07030-01	EU715179	Singapore	2007	
01_AE.SI.02.SI900_02	AJ971134	Slovenia	2002	
01_AE.SI.03.SI3493_03	AJ971102	Slovenia	2003	
01_AE.KR.06.BJH_003	EF157859	South Korea	2006	
01_AE.KR.07.K219	GQ290740	South Korea	2007	
01_AE.KR.08.K234	GQ290742	South Korea	2008	
01_AE.ES.07.07SP29_353349	EU255514	Spain	2007	
01_AE.ES.07.PN70	GQ241014	Spain	2007	

01_AE.SE.98.98SE_14962	AY165194	Sweden	1998
01_AE.SE.00.00SE_19382	AY165184	Sweden	2000
01_AE.SE.01.01SE_21427	AY165258	Sweden	2001
01_AE.SE.04.3jw3yAJwzxQlx7E	GQ399478	Sweden	2004
01_AE.CH.02.661673	JF769779	Switzerland	2002
01_AE.CH.03.1462731	JF769786	Switzerland	2003
01_AE.CH.05.3719226	JF769799	Switzerland	2005
01_AE.CH.06.9629529	JF769814	Switzerland	2006
01_AE.CH.07.13822372	JF769820	Switzerland	2007
01_AE.TW.07.21152	HQ657661	Taiwan	2007
01_AE.TW.08.26584	HQ657702	Taiwan	2008
01_AE.TW.08.27003	HQ657755	Taiwan	2008
01_AE.TW.08.27309	HQ657786	Taiwan	2008
01_AE.TW.08.27347	HQ657790	Taiwan	2008
01_AE.TW.09.35816	HQ657848	Taiwan	2009
01_AE.TW.09.35822	HQ657849	Taiwan	2009
01_AE.TW.09.35886	HQ657857	Taiwan	2009
01_AE.TW.09.36083	HQ657881	Taiwan	2009
01_AE.TW.09.36250	HQ657906	Taiwan	2009
01_AE.TH.90.90TH_CM244	AY713425	Thailand	1990
01_AE.TH.90.CM235	AF259954	Thailand	1990
01_AE.TH.90.CM240	U54771	Thailand	1990
01_AE.TH.91.CM238	AF447838	Thailand	1991
01_AE.TH.91.CM243	AF447828	Thailand	1991
01_AE.TH.93.93TH051	AB220944	Thailand	1993
01_AE.TH.93.93TH054	AB220945	Thailand	1993
01_AE.TH.93.93TH057	AB253424	Thailand	1993
01_AE.TH.93.93TH060	AB220946	Thailand	1993
01_AE.TH.93.93TH062	AB220947	Thailand	1993
01_AE.TH.93.93TH065	AB220948	Thailand	1993
01_AE.TH.93.93TH253	U51189	Thailand	1993
01_AE.TH.93.93TH9021	AF164485	Thailand	1993
01_AE.TH.94.94TH702	AF170545	Thailand	1994
01_AE.TH.94.94TH7091	AF170546	Thailand	1994
01_AE.TH.94.94TH7092	AF170547	Thailand	1994
01_AE.TH.94.POC30506	AF447817	Thailand	1994

01_AE.TH.95.95TNIH022	AB032740	Thailand	1995
01_AE.TH.95.95TNIH047	AB032741	Thailand	1995
01_AE.TH.95.NP1465	AF447827	Thailand	1995
01_AE.TH.96.96TH_M02138	AY713424	Thailand	1996
01_AE.TH.96.96TH_NI1046-01	AY713421	Thailand	1996
01_AE.TH.96.96TH_NI1149-01	AY713426	Thailand	1996
01_AE.TH.96.M114	DQ354117	Thailand	1996
01_AE.TH.97.97TH_NP1525	AY713420	Thailand	1997
01_AE.TH.97.97TH_NP1695	AY713419	Thailand	1997
01_AE.TH.97.97TH6_107	AY125894	Thailand	1997
01_AE.TH.98.98TH_NP1251	AY713422	Thailand	1998
01_AE.TH.98.98TH_R1166	AY945728	Thailand	1998
01_AE.TH.99.99TH_C1080	AY945712	Thailand	1999
01_AE.TH.99.99TH_C2405	AY945718	Thailand	1999
01_AE.TH.99.99TH_C4460	AY945726	Thailand	1999
01_AE.TH.99.99TH_NI1052	AY713423	Thailand	1999
01_AE.TH.99.99TH_R1149	AY945727	Thailand	1999
01_AE.TH.99.99TH_R3006	AY945731	Thailand	1999
01_AE.TH.99.99TH_R3265	AY945732	Thailand	1999
01_AE.TH.99.OUR044I	AY358042	Thailand	1999
01_AE.TH.99.OUR066I	AY358043	Thailand	1999
01_AE.TH.99.OUR098I	AY358044	Thailand	1999
01_AE.TH.99.OUR199I	AY358039	Thailand	1999
01_AE.TH.99.OUR422I	AY358051	Thailand	1999
01_AE.TH.00.00TH_C2101	AY945716	Thailand	2000
01_AE.TH.00.00TH_C2257	AY945717	Thailand	2000
01_AE.TH.00.00TH_C3347	AY945721	Thailand	2000
01_AE.TH.00.00TH_C4118	AY945722	Thailand	2000
01_AE.TH.00.00TH_C4151	AY945724	Thailand	2000
01_AE.TH.00.C1705	DQ789392	Thailand	2000
01_AE.TH.00.OUR595I	AY358052	Thailand	2000
01_AE.TH.00.OUR661I-01	AY358057	Thailand	2000
01_AE.TH.00.OUR721I-01	AY358067	Thailand	2000
01_AE.TH.00.OUR724I-01	AY358060	Thailand	2000
01_AE.TH.00.OUR746I-01	AY358061	Thailand	2000
01_AE.TH.00.OUR810I-01	AY358063	Thailand	2000

01_AE.TH.00.ThaiNIH01_C1w	AY961586	Thailand	2000
01_AE.TH.01.01TH_C1436	AY945713	Thailand	2001
01_AE.TH.01.01TH_C2570	AY945719	Thailand	2001
01_AE.TH.01.01TH_C3256	AY945720	Thailand	2001
01_AE.TH.01.01TH_R2184	AY945730	Thailand	2001
01_AE.TH.01.OUR414I	AY358050	Thailand	2001
01_AE.TH.01.OUR609I-01	AY358040	Thailand	2001
01_AE.TH.01.OUR642I-01	AY358041	Thailand	2001
01_AE.TH.01.OUR647I-01	AY358056	Thailand	2001
01_AE.TH.01.OUR702I-01	AY358059	Thailand	2001
01_AE.TH.01.OUR786I-01	AY358036	Thailand	2001
01_AE.TH.01.OUR788I-01	AY358068	Thailand	2001
01_AE.TH.01.OUR830I-01	AY358064	Thailand	2001
01_AE.TH.02.OUR769I-01	AY358062	Thailand	2002
01_AE.TH.03.PHPT1216PR_RT-01	HQ996502	Thailand	2003
01_AE.TH.04.04TH107542	JN248318	Thailand	2004
01_AE.TH.04.BKD	DQ314731	Thailand	2004
01_AE.TH.04.BKM	DQ314732	Thailand	2004
01_AE.TH.05.05TH127331	JN248338	Thailand	2005
01_AE.TH.06.PHPT2464PR_RT	HQ996508	Thailand	2006
01_AE.TH.06.PHPT4016PR_RT	HQ996504	Thailand	2006
01_AE.TH.06.PHPT4484PR_RT	HQ996505	Thailand	2006
01_AE.TH.07.PHPT2780PR_RT	HQ996506	Thailand	2007
01_AE.TH.07.PHPT2944PR_RT	HQ996503	Thailand	2007
01_AE.TH.07.PHPT4015PR_RT	HQ996509	Thailand	2007
01_AE.TH.07.PHPT4397PR_RT	HQ996500	Thailand	2007
01_AE.TH.07.PHPT4619PR_RT	HQ996507	Thailand	2007
01_AE.TH.08.PHPT2522PR_RT	HQ996499	Thailand	2008
01_AE.GB.07.77406	JQ361675	United Kingdom	2007
01_AE.US.98.98US_MSC1120	AY444803	United States	1998
01_AE.US.98.98US_MSC2008	AY444805	United States	1998
01_AE.US.98.98US_MSC3012	AY444806	United States	1998
01_AE.US.00.00US_MSC1164	AY444804	United States	2000
01_AE.US.03.03_108028	EU611711	United States	2003
01_AE.US.03.03_109722	EU611948	United States	2003
01_AE.US.03.03_120973	EU614130	United States	2003

01_AE.US.03.03_125736	EU615037	United States	2003	
01_AE.US.03.03_125737	EU615038	United States	2003	
01_AE.US.04.ARC-A25	EU711473	United States	2004	
01_AE.US.04.ARC-A32	EU711442	United States	2004	
01_AE.VN.97.97VNAG201	FJ185245	Vietnam	1997	IMC-1
01_AE.VN.97.97VNAG202	FJ185246	Vietnam	1997	
01_AE.VN.97.97VNAG204	FJ185247	Vietnam	1997	
01_AE.VN.97.97VNAG206	FJ185248	Vietnam	1997	
01_AE.VN.97.97VNAG207	FJ185249	Vietnam	1997	IMC-1
01_AE.VN.97.97VNAG210	FJ185251	Vietnam	1997	IMC-1
01_AE.VN.97.97VNAG212	FJ185252	Vietnam	1997	
01_AE.VN.97.97VNAG214	FJ185253	Vietnam	1997	IMC-1
01_AE.VN.97.97VNAG218	FJ185255	Vietnam	1997	IMC-1
01_AE.VN.97.97VNHCM301	FJ185237	Vietnam	1997	IMC-1
01_AE.VN.97.97VNHCM302	FJ185238	Vietnam	1997	IMC-1
01_AE.VN.97.97VNHCM303	FJ185239	Vietnam	1997	IMC-1
01_AE.VN.97.97VNHCM310	FJ185240	Vietnam	1997	IMC-1
01_AE.VN.97.97VNHCM314	FJ185241	Vietnam	1997	IMC-1
01_AE.VN.97.97VNHCM319	FJ185242	Vietnam	1997	
01_AE.VN.97.97VNHCM343	FJ185243	Vietnam	1997	
01_AE.VN.97.97VNHCM345	FJ185244	Vietnam	1997	IMC-1
01_AE.VN.98.98VNBG4	FJ185228	Vietnam	1998	IMC-1
01_AE.VN.98.98VNBG5	FJ185229	Vietnam	1998	IMC-1
01_AE.VN.98.98VNBG6	FJ185230	Vietnam	1998	IMC-1
01_AE.VN.98.98VNBG7	FJ185231	Vietnam	1998	IMC-1
01_AE.VN.98.98VNHD10	FJ185232	Vietnam	1998	IMC-1
01_AE.VN.98.98VNHD11	FJ185233	Vietnam	1998	IMC-1
01_AE.VN.98.98VNHD9	FJ185234	Vietnam	1998	IMC-1
01_AE.VN.98.98VNND15	FJ185235	Vietnam	1998	IMC-1
01_AE.VN.98.98VNND17	FJ185236	Vietnam	1998	IMC-1
01_AE.VN.04.VN_6	HQ245678	Vietnam	2004	IMC-1
01_AE.VN.05.VN_95	HQ245679	Vietnam	2005	
01_AE.VN.07.07VNHPA1	FJ007113	Vietnam	2007	IMC-1
01_AE.VN.07.07VNHPA13ag	FJ007116	Vietnam	2007	IMC-1
01_AE.VN.07.07VNHPA2_122	FJ007010	Vietnam	2007	IMC-1
01_AE.VN.07.07VNHPA2_177	FJ007026	Vietnam	2007	IMC-1

01_AE.VN.07.07VNHPA2_20	FJ006987	Vietnam	2007	IMC-1
01_AE.VN.07.07VNHPA2_56tt	FJ006993	Vietnam	2007	IMC-1
01_AE.VN.07.07VNHPA2_84ca	FJ007000	Vietnam	2007	IMC-1
01_AE.VN.07.07VNHPA35ga	FJ007121	Vietnam	2007	IMC-1
01_AE.VN.07.07VNHPA39c	FJ007124	Vietnam	2007	
01_AE.VN.07.07VNHPA57	FJ007131	Vietnam	2007	IMC-1
01_AE.VN.07.07VNHPC19	FJ006962	Vietnam	2007	IMC-1
01_AE.VN.07.07VNHPC39	FJ006969	Vietnam	2007	IMC-1
01_AE.VN.07.07VNHPC58a	FJ006976	Vietnam	2007	IMC-1
01_AE.VN.07.07VNHPC78ca	FJ006979	Vietnam	2007	IMC-1
01_AE.VN.07.07VNHPE14g	FJ006949	Vietnam	2007	IMC-1
