# Supplementary material for: Phylodynamic Analysis Reveals CRF01_AE Dissemination between Japan and Neighboring Asian Countries and the Role of Intravenous Drug Use in Transmission
Source: PLoS One. 2014 Jul 15;9(7):e102633. doi: 10.1371/journal.pone.0102633 (PMC4099140; doi:10.1371/journal.pone.0102633)
Supplement: Table S2 — Bayesian factor analysis of molecular clock models compared for constant demographic size. (PDF) [file pone.0102633.s008.pdf]

Table S2. Bayesian factor analysis of molecular clock models compared for constant demographic size.

Clock model	ln P(model | data)	S.E.	Strict	Lognormal	Exponential	Random
Strict	-28684.075	+/- 0.565	-	-65.35	-69.404	39.726
Lognormal relaxed	-28524.266	+/- 0.528	69.404	-	4.054	109.129
Exponential relaxed	-28533.601	+/- 0.548	65.35	-4.054	-	105.075
Random local	-28775.546	+/- 0.78	-39.726	-105.075	-109.129	-

Bayesian MCMC was not obtained in each demographic model other than constant size because the coalescent likelihood reached infinity.
