# Supplementary material for: Phylodynamic Analysis Reveals CRF01_AE Dissemination between Japan and Neighboring Asian Countries and the Role of Intravenous Drug Use in Transmission
Source: PLoS One. 2014 Jul 15;9(7):e102633. doi: 10.1371/journal.pone.0102633 (PMC4099140; doi:10.1371/journal.pone.0102633)
Supplement: Table S3 — Independence test of risk behaviors and nationalities in CRF01_AE-infected patients in Japan. (PDF) [file pone.0102633.s009.pdf]

Table S3. Independence test of risk behaviors and nationalities in CRF01_AE-infected patients in Japan.

	Japanese	Foreign	odds ratio (OR)	p-value
Heterosexual	116	44	0.556	0.183
Other risks	38	8		
MSM	36	1	15.444	0.0001
Other risks	118	51		
IVDU	2	7	0.086	0.0011
Other risks	152	45		

Patients with unknown risk behaviors or nationalities were excluded from the analysis.
