# Supplementary material for: Phylodynamic Analysis Reveals CRF01_AE Dissemination between Japan and Neighboring Asian Countries and the Role of Intravenous Drug Use in Transmission
Source: PLoS One. 2014 Jul 15;9(7):e102633. doi: 10.1371/journal.pone.0102633 (PMC4099140; doi:10.1371/journal.pone.0102633)
Supplement: Table S4 — Estimates of the mean evolutionary diversity for categories of CRF01_AE sequences. (PDF) [file pone.0102633.s010.pdf]

Table S4. Estimates of the mean evolutionary diversity for each group of CRF01_AE sequences.

	Mean inter-population diversity (net)		Coefficient of differentiation	
	d	S.E.	d	S.E.
Inter-patients in Japan	0.0328	0.0025	-	-
CRF01_AE Asian variant	0.0341	0.0025	-	-
Within reconstructed clones	0.0012	0.0002	-	-
Between reconstructed clones	0.0393	0.0027	0.9642	0.0056
Within risk behaviors	0.0371	0.0063	-	-
Between risk behaviors	0.0017	0.0020	0.0357	0.0380
Within collection areas	0.0319	0.0023	-	-
Between collection areas	0.0058	0.0009	0.1541	0.0213
Within micro-clades	0.0097	0.0007	-	-
Between micro-clades	0.0296	0.0021	0.6054	0.0137
