# Supplementary material for: Phylodynamic Analysis Reveals CRF01_AE Dissemination between Japan and Neighboring Asian Countries and the Role of Intravenous Drug Use in Transmission
Source: PLoS One. 2014 Jul 15;9(7):e102633. doi: 10.1371/journal.pone.0102633 (PMC4099140; doi:10.1371/journal.pone.0102633)
Supplement: Table S5 — Evolutionary parameters obtained in Bayesian MCMC inference with constant size and lognormal relaxed. (PDF) [file pone.0102633.s011.pdf]

Table S5. Evolutionary parameters obtained in Bayesian MCMC inference with constant size and lognormal relaxed clock models.

Parameter	Mean	95% HPD L	95% HPD H
Mean rate	1.07×10^{-3}	0.59×10^{-3}	1.91×10^{-3}
Coefficient of variation	0.597	0.514	0.686
Likelihood	-40415.04	-40539.91	-40321.34
Population size	3.03×10^{5}	2.62×10^{5}	3.46×10^{5}

Evolutionary rate is represented in per site per year.
